# Engineering Cobalt Bis(dicarbollide)-Loaded Polymeric Nanocarriers: From Controlled Fabrication to Drug Delivery for BNCT

**DOI:** 10.3390/jfb17080403

**Published:** 2026-08-14

**Authors:** Maksim A. Klimenko, Maria B. Sokol, Mariia R. Mollaeva, Nikita G. Yabbarov, Ivan A. Gulyaev, Margarita V. Chirkina, Olga E. Kamaeva, Igor B. Sivaev, Anna A. Druzina, Vladimir I. Bregadze, Irina Yu. Nikolaeva, Andrey Yu. Bychkov, Vladimir A. Khvostikov, Zhanna P. Burmii, Sergei I. Obydennyi, Elena D. Nikolskaya

**Affiliations:** 1 N.M. Emanuel Institute of Biochemical Physics, Russian Academy of Sciences, 119334 Moscow, Russia; 2 A.N. Nesmeyanov Institute of Organoelement Compounds, Russian Academy of Sciences, 119334 Moscow, Russia; sivaev@ineos.ac.ru (I.B.S.); ilinova_anna@mail.ru (A.A.D.); bre@ineos.ac.ru (V.I.B.); 3 Faculty of Geology, Lomonosov Moscow State University, GSP-1, Leninskie Gory, 119991 Moscow, Russia; niko-geo@mail.ru (I.Y.N.); andrewbychkov@rambler.ru (A.Y.B.); 4 The Institute of Microelectronics Technology and High-Purity Materials, Russian Academy of Sciences, 142432 Chernogolovka, Russia; vkhvostikov@mail.ru (V.A.K.); burmii@iptm.ru (Z.P.B.); 5 Dmitry Rogachev National Medical Research Center of Pediatric Hematology, Oncology and Immunology, 117198 Moscow, Russia; 6 Center for Theoretical Problems of Physicochemical Pharmacology, 119334 Moscow, Russia

**Keywords:** nanoparticles, nanocarriers, PLGA, COSAN, Box–Behnken design, controlled release, design of experiment, boron neutron capture therapy

## Abstract

Poly(lactic-co-glycolic) acid nanoparticles (NPs) with the addition of the sodium salt of cobalt bis(1,2-dicarbollide) (COSAN) were developed for boron neutron capture therapy (BNCT). The Box–Behnken design was applied to predict an optimal value of variables and to obtain the desired NP characteristics. The optimized NPs had a size of 166 nm and a COSAN loading of 16%. Based on FTIR and UV-VIS spectrophotometry data, it was found that COSAN maintains chemical stability during the encapsulation process and exhibits prolonged release from NPs. A study of in vitro cytotoxicity and accumulation of the NPs on B16 cells revealed their low toxicity and high accumulation in cells after 24 h. In vivo analysis in B16-bearing BALB/c nude mice showed preliminary tumor-associated ^1^^0^B distribution 12 h after iv administration of a deliberately low NP-CPPC dose (50 µg per mouse). The estimated intratumoral concentration was 0.31 µg ^1^^0^B/g, which is below commonly cited BNCT delivery benchmarks. These results show high potential of the formulation, but require further optimization of dose, timing, biodistribution, normal-tissue exposure, tolerability and therapeutic-dose BNCT evaluation.

## 1. Introduction

Cancer remains a global health and socioeconomic issue in the 21st century, accounting for approximately one-sixth of all mortality and a quarter of fatalities attributed to noncontagious diseases worldwide [1]. Although surgery, radiation therapy, and chemotherapy have made significant advancements, the challenge of effectively treating patients with malignant tumors such as glioblastoma multiforme, gliomas, melanomas, and their metastases remains unresolved. This is primarily because these treatment methods often harm both cancerous and healthy tissues in the affected areas.

Among emerging oncological strategies, boron neutron capture therapy (BNCT), a form of neutron capture therapy (NCT), attracted considerable interest as a targeted approach for malignancies that are inaccessible or respond inadequately to standard therapeutic interventions. Clinical trials on BNCT efficacy have shown positive results in the treatment of glioblastoma, melanoma, neck tumor, meningioma, pleural mesothelioma and hepatocellular carcinoma [2,3].

The therapeutic principle of BNCT relies on the interaction between two components: thermal neutrons and a boron-containing agent capable of selectively delivering the stable isotope ^1^^0^B to tumor cells. The ^1^^0^B nucleus possesses a high thermal neutron capture cross-section, and upon neutron absorption undergoes the intracellular nuclear reaction ^1^^0^B(n,α) ^7^Li. This reaction generates two high linear energy transfer (LET) particles—the α-particle ^4^He^2^^+^ and the lithium recoil ion ^7^Li^3^^+^, along with a 480 keV photon. The combined kinetic energy released (2.4 MeV) is deposited over a path length of approximately 5–9 µm in biological tissue, a distance comparable to the diameter of a single cell. As a result, the cytotoxic effect is spatially confined to the cell in which the nuclear reaction occurs, such that selective ^1^^0^B accumulation within tumor cells, followed by neutron irradiation, is expected to achieve targeted tumor cell destruction with minimal collateral damage to adjacent healthy tissue [4,5].

**Figure 1:**
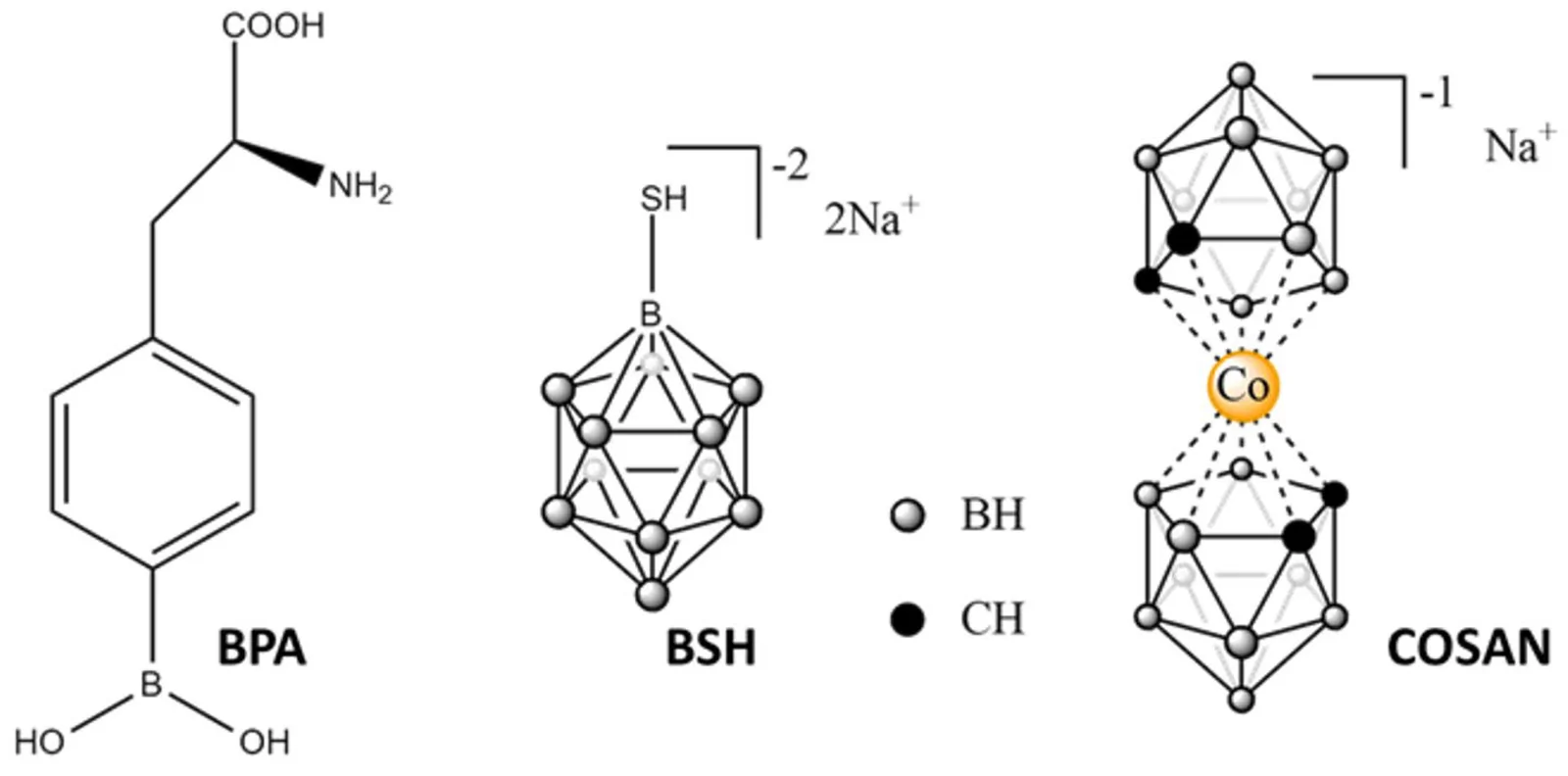
Structures of drugs for BNCT.

To date, two boron-containing compounds are employed in clinical BNCT trials: L-boronophenylalanine (BPA) and sodium borocaptate (BSH) [6] (Figure 1). Despite demonstrating clinical efficacy in BNCT-treated patients, neither agent fully satisfies the criteria established for an ‘ideal’ boron delivery agent [7]. This drives chemists and pharmacists to design new promising boron delivery agents. Among them is cobalt bis(dicarbollide) anion (COSAN), a polyhedral boron hydride containing 18 boron atoms per molecule [8,9,10]. Due to excellent stability, COSAN is attractive for encapsulation into various drug delivery systems to enhance boron accumulation into tumor cells [11,12,13].

In the present study, COSAN-loaded nanoparticles (NPs) were developed using poly(lactic-co-glycolic acid) (PLGA) as the polymeric matrix. In our study, we referred to colloidal polymeric particles ranging in size from 10 to 1000 nm as nanoparticles according to the IUPAC [14] and the Encyclopedia of Pharmaceutical Technology [15] definition. PLGA is a well-established polymer for drug delivery applications owing to its favorable biocompatibility, biodegradability, and capacity to enhance drug accumulation within tumor tissue [16]. Tumor accumulation of PLGA nanoparticles is governed by several complementary mechanisms. Passive accumulation occurs primarily via the enhanced permeability and retention (EPR) effect, which originates from the aberrant architecture and increased vascular permeability of tumor tissue combined with impaired lymphatic drainage; these features allow nanoparticles in the 50–250 nm size range to preferentially extravasate and accumulate within the tumor microenvironment [17]. Following extravasation, cellular internalization proceeds predominantly through energy-dependent endocytic pathways including clathrin- and caveolae-mediated endocytosis and micropinocytosis, with the partial contribution of each pathway determined by nanoparticle size, surface characteristics, and cell type [18]. In addition, surface functionalization with targeting ligands can further enhance nanoparticle uptake via receptor-mediated endocytosis, thereby improving tumor cell selectivity and intracellular delivery of the therapeutic payload [19,20].

**Figure 2:**
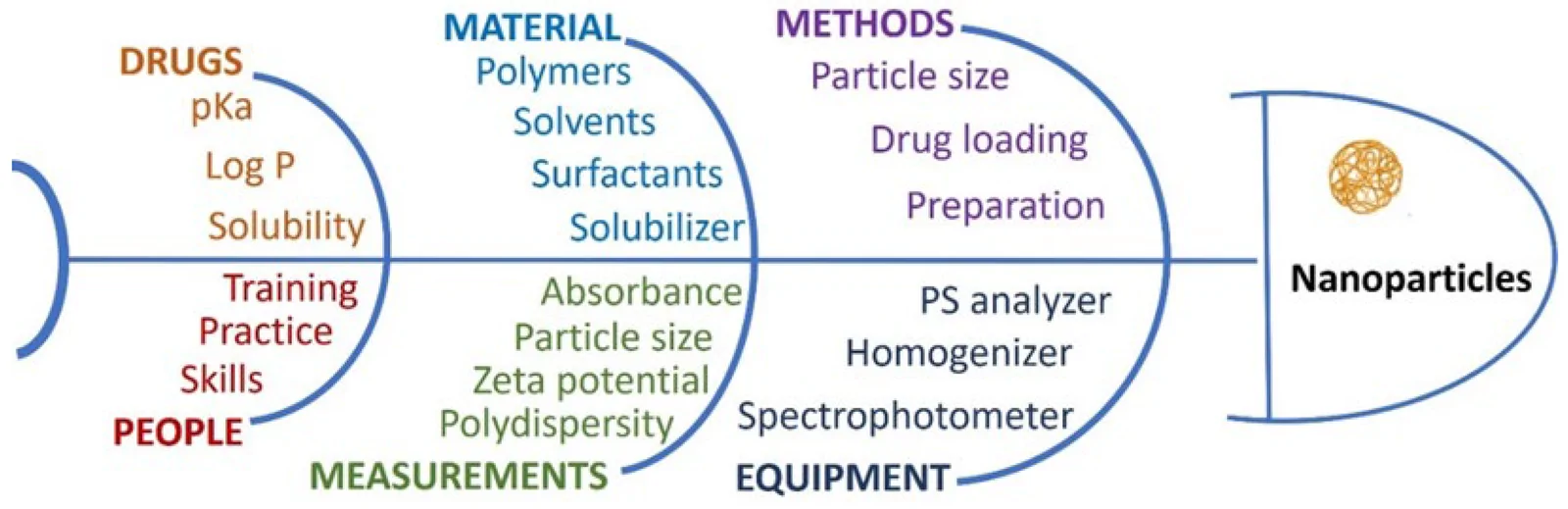
Ishikawa diagram describing the factors influencing NP design.

The application of PLGA nanoparticles as polymeric carriers for carborane-based BNCT agents has been documented [21,22,23]. The design of NPs is a complex process since any modification of the conditions during NP synthesis affects their physicochemical characteristics, biological parameters, and pharmacological activity (Figure 2).

PLGA provides a biodegradable hydrophobic matrix suitable for incorporation of lipophilic boron derivatives; however, unmodified hydrophobic nanoparticles can undergo adsorption of plasma proteins and rapid uptake by the mononuclear phagocyte system. Incorporation of a PLGA–PEG copolymer into a PLGA matrix is therefore a rational strategy to create a PEG-rich hydrophilic interfacial layer. Such a layer can reduce nonspecific protein adsorption, improve colloidal stability, and prolong circulation of polymeric nanoparticles, thereby increasing the opportunity for tumor exposure [24,25,26]. At the same time, the effect of PEG depends on its molecular weight, surface density, particle size, and formulation composition; excessive PEG shielding can hinder cell–particle interactions [27,28]. Consequently, the PLGA/PLGA–PEG ratio must be optimized empirically rather than assumed to be universally beneficial [29,30]. Previous boron nanomedicine studies have demonstrated the potential of polymeric carriers but also showed some limitations: carborane-loaded, PSMA-targeted PEG-b-PLGA nanoparticles were developed for theranostic and prostate-cancer targeting; in a melanoma study, o-carborane was encapsulated into PLGA nanoparticles, demonstrating that polymer composition can markedly influence burst release and tumor boron accumulation [21,22,31]. These studies reveal the potential of polymeric boron delivery but do not remove the need to balance the interplay among polymer composition, emulsifier concentration, organic-phase conditions, particle size, and boron loading for each boron derivative and formulation method.

Response surface methodology offers a complex approach to this optimization problem. The Box–Behnken design (BBD) is a three-level experimental approach that estimates linear, quadratic, and interaction effects among formulation variables using substantially fewer experiments than a full three-level factorial design. It is well suited to nanoparticle development, where changing one variable at a time cannot reveal interactions among polymer amount, stabilizer concentration, and organic-phase volume [29,32,33,34,35]. BBD was applied to PLGA nanoparticle formulations to jointly optimize particle size, dispersity, surface charge, and drug-loading-related responses [36]. In our study, BBD was used to quantify the effects and interactions of PLGA amount, PVA concentration, and chloroform volume on the size and COSAN loading of the nanoparticles and, finally, to develop a model-enabled selection of a reproducible formulation rather than a formulation chosen empirically. This formulation-oriented approach distinguishes the present work from prior studies focused on a single PLGA/PLLGA matrix, covalently functionalized PEG-b-PLGA targeting systems, or non-systematically optimized carborane carriers.

Previously, we have successfully applied the BBD for the development of metalloporphyrins-loaded polymeric NPs [37] and for optimization of PLGA NP analysis by high-performance liquid chromatography [38]. The BBD approach provides applied statistics to evaluate the factors that control the value of a parameter, yielding effective optimization of the NP design process.

The aim of this study was to develop novel COSAN-loaded PLGA NPs via the BBD approach for BNCT.

## 2. Materials and Methods

### 2.1. Materials

Acid-terminated poly(D,L-lactide-co-glycolide) (PLGA) with a monomer ratio of 50:50, a molecular weight of 17–21 kDa, and an inherent viscosity of 0.2 dL/g in hexafluoroisopropanol (HFIP) was obtained from LACTEL Absorbable Polymers (Birmingham, AL, USA). D-Mannitol, poly(vinyl alcohol) (PVA, MW 30,000–70,000), NH_2_-PEG(12)-NH-Boc, N-Hydroxysuccinimide (NHS), N,N-diisopropylethylamine (DIPEA) and N-butyl alcohol were purchased from Sigma-Aldrich (St. Louis, MO, USA). 1-Ethyl-3-(3-dimethylaminopropyl)carbodiimide (EDC) was purchased from Fluka (St. Gallen, Switzerland). Dimethyl sulfoxide (DMSO), chloroform, and methylene chloride were purchased from Ruskhim (Moscow, Russia). Tween 80 was purchased from Serva (Heidelberg, Germany). Diethyl ether was purchased from Khimmed Syntes (Moscow, Russia). Deuterochloroform (CDCl_3_) was purchased from Cambridge Isotope (Canada, Andover, UDA). Cyanine5.5 (Cy5.5) was purchased from (Lumiprobe, Moscow, Russia). Sodium dodecyl sulfate (SDS) was purchased from PanReac AppliChem (Chicago, IL, USA).

The following were also purchased: DMEM, FBS, gentamicin, 3-[4,5-dimethylthiazolyl-2-yl]-2,5-diphenyltetrazoliumbromide (MTT), mowiol, agarose, sodium chloride, tris(hydroxymethyl)aminomethane hydrochloride, phenyl methyl sulphonyl fluoride, sodium dodecyl sulfate, Hoechst 34580 and sodium deoxycholate (Sigma-Aldrich, St Louis, MO, USA); phosphate-buffered saline (PBS) (Amreso, Solon, OH, USA); trypsin (Hyclone, Logan, UT, USA); and ethylenediaminetetraacetic acid (EDTA), L-glutamine, Triton X-100 and paraformaldehyde (PFA) (Serva, Heidelberg, Germany).

The sodium salt of cobalt bis(dicarbollide) Na [3,3′-Co(1,2-C_2_B_9_H_11_)_2_] (COSAN) was synthesized as previously described [39,40]. PLGA-PEG was synthesized as previously described [41].

### 2.2. Cell Culture

B16 murine melanoma cells (ATCC, Manassas, VA, USA) were maintained in DMEM supplemented with 10% FBS, 2 mM L-glutamine and 50 µg/mL gentamicin, cultured under standard conditions (humidified atmosphere, +37 °C, 5% CO_2_) and harvested using 0.05% trypsin- 0.02% EDTA mix before reaching 80% confluence.

### 2.3. Animals

Athymic BALB/c nude female mice (15–20 g) (Pushchino Animal Breeding Facility, Pushchino, Russia) were housed in a vivarium under a natural light cycle with access to standard pelleted feed and water *ad libitum*. All experimental procedures were conducted in accordance with the Order of the Ministry of Health of the Russian Federation No. 199 (1 April 2016), on the Approval of the Rules for GLP, and with the principles of the World Medical Association (WMA) Declaration of Helsinki.

### 2.4. Preparation of Nanoparticles

#### 2.4.1. Box–Behnken Experimental Design

The NP formulation was optimized using a BBD with response surface methodology [42]. A 2-level, 3-factor design with 5 central-point replicates was implemented, comprising 12 experimental runs in total [43]. The independent variables were PVA concentration (A, %), PLGA amount (B, mg), and CHCl_3_ volume (C, mL), and their effects on particle size (R1, nm) and drug loading (DL, R2, %) were assessed at low and high levels as defined in Table 1.

Statistical analysis using Design-Expert software (v. 7.0; Stat-Ease Inc., Minneapolis, MN, USA) was conducted to determine the relative impact of each factor, factor interactions, and associated statistical values. Numerical optimization was subsequently performed to identify formulation conditions that simultaneously maximized DL and minimized particle size. The solution with the highest desirability score was selected, and the corresponding optimized nanoparticles were prepared and characterized; experimentally observed values were then compared against model predictions.

#### 2.4.2. Preparation of COSAN-Loaded NPs (COSAN-NP)

We prepared the NPs using a double emulsion-solvent evaporation method, which is well suited for the encapsulation of water-soluble compounds. Quantities are given for obtaining an optimized batch of nanoparticles after a Box–Behnken experimental design (BBD). For BBD, the quantities used in the method are as indicated in Table 2.

Briefly, 10 mg of COSAN was dissolved in 0.554 mL of distilled water, and 10 mg of PLGA was dissolved in 2.77 mL of chloroform to form the organic phase. The COSAN solution was then added to the PLGA solution and homogenized using an ULTRA-TURRAX T25 basic disperser (IKA Werke, Germany) at 24,000 rpm for 40 s; this step was repeated four times with 20 s intervals between cycles. The resulting primary emulsion was added dropwise to 13.85 mL of 0.33% (*w*/*v*) PVA solution and stirred for 10 min. The mixture was subsequently sonicated using a Labsonic U ultrasonic homogenizer (B. Braun, Melsungen, Germany) in High+ mode with a repeating duty cycle of 0.9, in an ice bath; sonication was performed four times for 40 s with 20 s intervals between cycles. The organic solvent was rapidly removed under reduced pressure using a Hei-VAP Advantage G3B ML rotary evaporator (Heidolph, Germany) at 37 °C. The resulting NP suspension was centrifuged at 32,000× *g* for 30 min at 4 °C (Beckman J2-2, Palo Alto, CA, USA), and the pellet was resuspended in 5.0 mL of distilled water. The suspension was filtered through a glass filter (pore size 111 µm), supplemented with 10% (*w*/*w*) D-mannitol as a cryoprotectant, lyophilized (Alpha 1-5, Christ, Germany), and stored at 4 °C until further use.

#### 2.4.3. Preparation of COSAN-Loaded PLGA/PLGA-PEG NPs (NP-CPP)

First, 10 mg of COSAN was dissolved in 0.554 mL of distilled water. As a polymeric matrix, 10 mg of PLGA-COOH 50/50 and PLGA-NH-PEG(12)-NH_2_ mixture was used with the ratios of 0:100, 20:80, 40:60, 60:40, 80:20, and 100:0 (*w*/*w*,%), respectively. The polymeric mixtures were dissolved in 2.77 mL of CHCl_3_. After complete dissolution of the substance and the polymeric mixtures, the solutions were combined and stirred on a magnetic stirrer for 10 min. Then nanoparticles were prepared according to Section 2.4.2.

#### 2.4.4. Preparation of Cy5.5 and COSAN-Loaded PLGA/PLGA-PEG NPs (NP-CPPC)

First, 2.5 mg of Cy5.5 was dissolved in 1 mL of CHCl_3_. Next, 6 mg of PLGA-PEG and 4 mg of PLGA were dissolved in 1.77 mL of CHCl_3_ (PLGA/PLGA-PEG ratio was 40/60). After complete dissolution of the substances, the solutions were mixed and stirred on a magnetic stirrer for 10 min. Then nanoparticles were prepared as described in Section 2.4.2.

### 2.5. Characterization of NPs

#### 2.5.1. Particle Size (PS), Zeta Potential, and Polydispersity Index (PDI)

PS, PDI, and the zeta potential of the NPs were determined by dynamic light scattering (DLS) and electrophoretic light scattering, respectively, using a Zetasizer Nano-ZS ZEN 3600 instrument (Malvern Instruments, Worcestershire, UK). Prior to measurement, samples were diluted with distilled water to a final concentration of 1 mg/mL, and three independent samples were analyzed for each formulation.

#### 2.5.2. DL Measurement

The drug loading (DL) of the NPs was quantified by inductively coupled plasma mass spectrometry (ICP-MS) and UV–Vis spectrophotometry. For the physicochemical analysis of NPs, UV-vis spectroscopy was used; for the quantitative determination of the boron isotope content in biological samples (cells and organs), the ICP-MS method was used.

UV-vis spectrophotometry was carried out with Spectronic Helios Alpha (Thermo Fisher Scientific, Waltham, WA, USA) by dissolving 5.0 mg of lyophilized NPs in 1 mL of DMSO. The analysis was performed at 284 nm. DL of obtained nanoparticles was calculated according to Equation (1):

DL = [(A·Mw·V)/(ε·l·m)]·100%,(1)

where m is the COSAN-loaded NPs mass (g), V is the DMSO volume (L), Mw is the COSAN molar mass equal to 344.71 g/mol, l is the optical path length (1 cm), ε is the extinction coefficient of COSAN in DMSO, equal to 82.22 L·mol^−1^·cm^−1^, and A is the absorbance of COSAN in DMSO.

ICP-MS analysis was performed on an Agilent 7500C (Agilent Technologies, Tokyo, Japan) equipped with a Babington nebulizer and a Peltier-cooled Scott spray chamber (2 °C). Data acquisition and processing were carried out using ICP-MS ChemStation software (version G1834B; Agilent Technologies, Santa Clara, CA, USA). Both samples and standard solutions were diluted in a 0.1% Triton X-100/1% HNO_3_ mixture, which also served as the carrier solution for the mass spectrometer.

#### 2.5.3. Transmission Electron Microscopy (TEM)

The morphology of the NPs was examined on a Tecnai Osiris instrument (FEI, Hillsboro, OR, USA). For sample preparation, a drop of NP aqueous suspension (1 mg/mL) was deposited onto a 3 mm copper grid coated with Formvar film and allowed to dry for 30 min at 25 °C.

#### 2.5.4. FTIR Analysis

The sample suspensions were compressed under a pressure of 10 t/nm^2^ to obtain KBr disks. The samples were scanned in the range from 400 to 4000 cm^−1^ on a Nicolet 380 instrument (Thermo Fisher Scientific Inc., USA) at a resolution of 4 cm^−^^1^ for a scan time of 30 s.

### 2.6. Drug Release Kinetics

First, 27.0 mg of NPs were resuspended in 35.0 mL of freshly prepared 0.01 M PBS pH 7.4 containing 0.1% Tween-80 (*w*/*v*). The resulting suspension was aliquoted into 1.5 mL centrifuge tubes followed by incubation at +37 °C and constant horizontal shaking. At predominant time intervals (0 min, 15 min, 30 min, 1 h, 4 h, 6 h, 30 h, 78 h, 211 h), the samples were centrifuged with an Eppendorf 5417 R centrifuge (Eppendorf, Germany) for 5 min at 20,800 *g* at +37 °C. The supernatant was withdrawn, and the pellet was lyophilized for 24 h. The resulting lyophilizate was analyzed by UV-vis spectrophotometry as described in Section 2.5.2. Data processing was carried out with DDsolver^®^ add-in for Microsoft Excel.

### 2.7. Hemolytic Activity

Hemolytic activity was assessed according to the published protocol with minor modifications [44]. Briefly, 4 mL of rabbit blood was collected from the ear vein and centrifuged at 358× *g* for 5 min at 4 °C. The resulting pellet was washed three times with PBS (0.01 M, pH 7.4) and resuspended in 13 mL of the same buffer. Blank NPs and COSAN-NPs at concentrations of 4, 0.4, and 0.04 mg/mL (250 µL each) were mixed with 250 µL of the red blood cell (RBC) suspension in 1.5 mL microtubes. The mixtures were incubated at 37 °C under continuous agitation for 3 h and then centrifuged at 2240× *g* for 5 min at room temperature. Aliquots of 250 µL from each supernatant were transferred to a 96-well plate, mixed with 3 µL of 6% SDS, and incubated for 5 min to convert hemoglobin to its low-spin oxidized form, hemichrome. The absorbance of the resulting solutions was measured at 540 nm to quantify hemoglobin release. RBCs treated with 10% Triton X-100 or 0.01 M PBS served as positive and negative controls, respectively. The percentage hemolysis was calculated according to Equation (2):

(2) $$Hemolysis\left(\%\right)=\frac{Abs\;sample-Abs\;negative}{Abs\;positive-Abs\;negative}\times 100\%$$

### 2.8. MTT Assay

B16 cells were seeded in a 96-well plate (4 × 10^3^ cells per well). Then, 24 h later, cells were treated with COSAN and NP-CPPC at the concentration range of 0.6–80.0 µM (in terms of COSAN) for 72 h. Cell viability was assessed using the MTT assay as previously described [37].

### 2.9. Analysis of NP-CPPC Accumulation Using Flow Cytometry

B16 cells were cultured in 3.5 cm Petri dishes (2×10^5^ cells per dish) in 1.5 mL of DMEM. The next day, cells were incubated with 50 μM NP-CPPC (in terms of COSAN) for 1 h, 2 h, 4 h and 24 h under standard conditions. Next, cells were harvested (425 g for 5 min at +4 °C), washed twice with 1 mL of PBS, and analyzed by flow cytometry (Cytoflex, Beckman, USA) (λ_ex_ 635 nm, λ_em_ 665 nm).

### 2.10. Analysis of NP-CPPC Accumulation Using Confocal Laser Scanning Microscopy

B16 cells were seeded in a 24-well plate with coverslips (12 mm) (15·10^3^ cells per well). After 24 h, the attached cells were treated with 50 μM NP-CPPC (in terms of COSAN) for 1 h, 2 h, 4 h and 24 h under standard conditions. Further, cells were rinsed with PBS and stained with 5 μg/mL Hoechst 34580 for 10 min, rinsed 3 times with PBS and fixed with 2% PFA for 24 h at +4 °C. After fixation, cells were washed twice with PBS and embedded in mowiol. The obtained samples were analyzed by Carl Zeiss Cell Observer Z1 CLSM (Carl Zeiss, Yen, Germany) with a 100χ magnification. The pictures were processed by Photoshop software 27.0 (Adobe Inc., San Jose, CA, USA).

### 2.11. Analysis of COSAN and NP-CPPC Accumulation Using Inductively Coupled Plasma Mass Spectrometry

B16 cells were seeded in 6 cm Petri dishes (1 × 10^6^ cells per dish) in 5 mL of DMEM. Then, 24 h later, cells were treated with 50 μM COSAN and 50 μM NP-CPPC (COSAN equivalent) for 1 h, 2 h, 4 h and 24 h under standard culture conditions. After incubation, cells were harvested (425× *g* for 7 min at 4 °C), then washed twice with 1 mL of PBS. Cell pellets were lysed in 100 µL of RIPA buffer (25 mM Tris-HCl, pH 7.4; 1% sodium deoxycholate (*w*/*v*); 1% Triton X-100 (*v*/*v*); 5 mM EDTA; 150 mM NaCl; 1 mM PMSF; 0.1% SDS (*w*/*v*)) for 30 min on ice. Cell lysates were then clarified by centrifugation at 15,300× *g* for 20 min at 4 °C, and the supernatants were analyzed by ICP-MS.

### 2.12. Analysis of NP-CPPC Accumulation in Tumor Spheroids

Agarose micro-well plates for spheroids were obtained as previously described [45]. B16 cells were seeded on micro-well plates in a 12-well plate at a cell density of 2·10^5^ cells/mL and incubated under standard conditions for 7 d. The culture plate was incubated under standard conditions, and the medium was changed every 2 days. After that, 3D cultures of B16 cells were incubated with 50 μM NP-CPPC (in terms of COSAN) for 1 h, 2 h, 4 h and 24 h. Tumor spheroid images were photographed by the JuLI™ Stage imaging system (Nanoentek Inc., Seoul, Republic of Korea).

### 2.13. In Vivo Biodistribution

A subcutaneous B16 tumor model was established by subcutaneous inoculation of B16 cells into a BALB/c nude mouse (10^5^ cells per mouse). After the tumor size reached approximately 500 mm^3^, 50 µg of NP-CPPC were intravenously injected in 100 µL of PBS. The mice were euthanized 12 h after injection, followed by collection of tumors and organs. In vivo and ex vivo visualization was performed prior to euthanasia using the FOBI CellGentek system (NeoScience/CellGentek, Nanoentek Inc., Seoul, Republic of Korea). After visualization, the collected organs were dissolved using an autoclave decomposition system with resistive heating ETAS-6 developed in IMT RUS (Russia). Samples weighing 50–350 mg were placed in PTFA reaction chambers, where 0.5 to 1.2 mL of HNO_3_ (nitric acid 65%; max. 0.0000005% Hg; GR, ISO, Merck, Darmstadt, Germany) was added depending on the mass; the samples were sealed in titanium autoclaves. The titanium autoclaves were placed in a thermostat and heated for 1 h at 180 °C and 1 h at 200 °C. After cooling, the autoclaves were opened, and the obtained solutions were transferred from the reaction chambers into 15 mL plastic test tubes. To the obtained solutions of 6 to 12 mL (depending on the sample weight) Sc was added as an internal standard; boron content was measured using an ICP-MS spectrometer XSeries II (Thermo Scientific, Waltham, WA, USA). The INCT-PVTL-6 (Polish Virginia Tobacco Leaves) standard developed in the Institute of Nuclear Chemistry and Technology (Poland) was used to control the correctness of the analysis procedure.

### 2.14. Statistical Analysis

All data are presented as mean ± S.D. unless otherwise stated. Statistical analyses were performed using Design-Expert (version 7.0; Stat-Ease Inc., Minneapolis, MN, USA), Data Acquisition Station (DAS; version 2.0; MREL Group of Companies, Kingston, ON, Canada), and OriginPro (version 2019b; OriginLab Corporation, Northampton, MA, USA). Group comparisons were conducted using Student’s *t*-test or one-way ANOVA, as appropriate. A *p*-value of <0.05 was considered statistically significant.

## 3. Results

### 3.1. Formulation and Optimization of COSAN-Loaded PLGA NPs (COSAN-NP)

**Figure 3:**
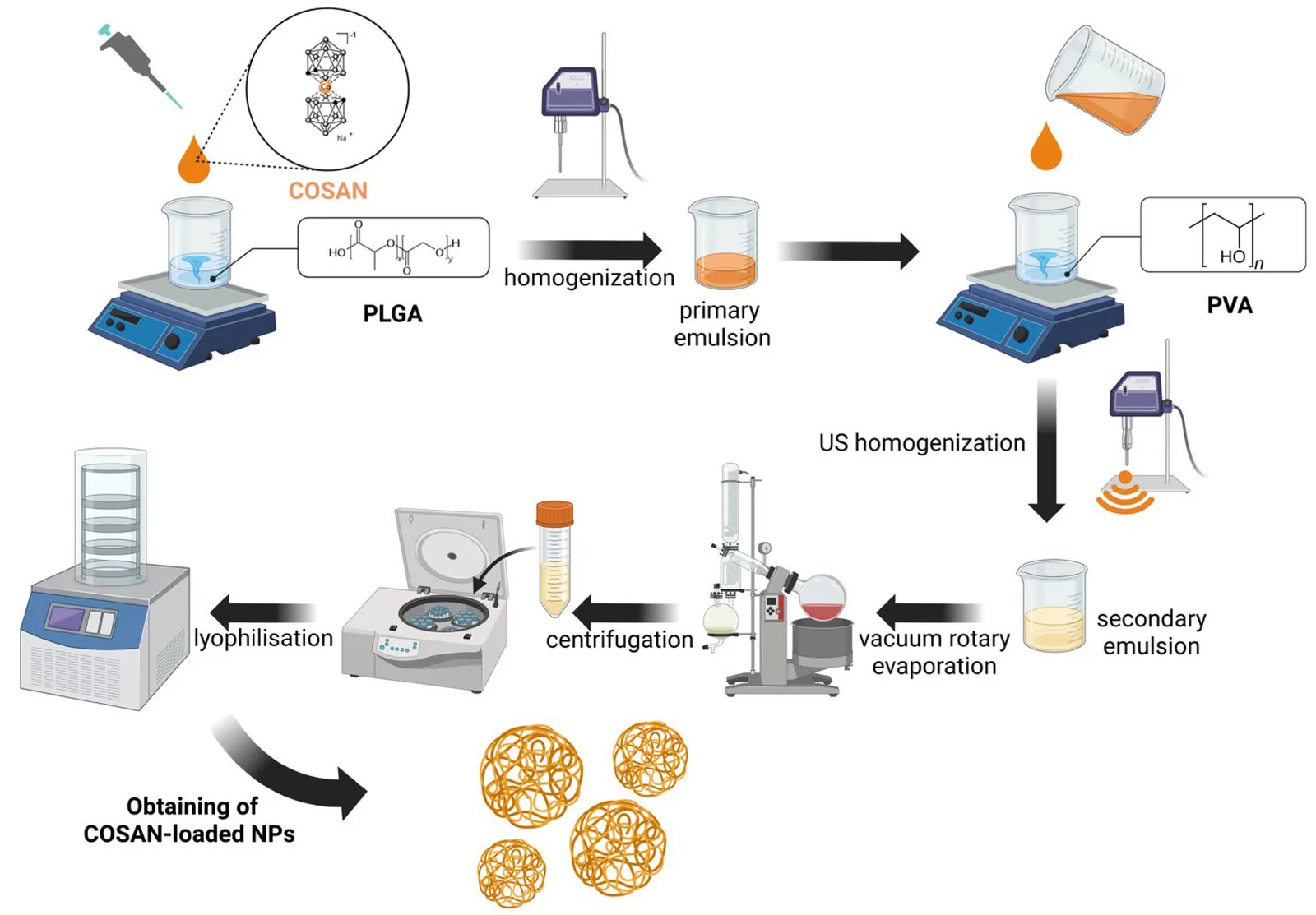
Schematic representation of COSAN-NPs formulation.

COSAN-loaded PLGA nanoparticles were prepared via double emulsion-solvent evaporation technique using mechanical homogenization to obtain the primary emulsion and ultrasound homogenization to obtain double emulsion (Figure 3).

A total of 12 experiments were performed with five central points (Table 2).

**Table 2:** Box–Behnken design matrix with observed responses for each randomized experimental run.

Experiment	Factors			Responses	
	A: PLGA (mg)	B: PVA (%)	C: CHCl_3_ (mL)	PS (nm)	DL (%)
1	32.50	0.75	2.50	342	2.2
2	55.00	0.50	7.50	230	1.0
3	10.00	0.25	5.00	220	2.6
4	55.00	0.50	2.50	220	2.6
5	10.00	0.50	7.50	396	1.4
6	32.50	0.25	7.50	342	1.5
7	10.00	0.50	2.50	220	3.3
8	10.00	0.75	5.00	220	1.9
9	55.00	0.75	5.00	255	1.0
10	32.50	0.25	2.50	180	3.5
11	32.50	0.75	7.50	255	1.1
12	55.00	0.25	5.00	164	1.8

Applied variable factors provided a wide distribution of particle size (R1; from 164 nm to 396 nm). Oppositely, the range of DL value was relatively narrow (R2; from 1.0% to 3.5%). Next, an experimental relationship between the mathematical model and the variables was established via polynomial Equations (3) and (4):

DL = 2.27 − 0.37·A − 0.39·B − 0.83·C − 0.05·A·B + 0.093·A·C + 0.22·B·C − 0.22·A^2^ − 0.24·B^2^, (3)

PS = 334.00 − 23.38·A + 19.50·B + 31.38·C + 22.75·A·B − 41.50·A·C − 59.75·B·C − 67.50·A^2^ − 51.75·B^2^, (4)

**Figure 4:**
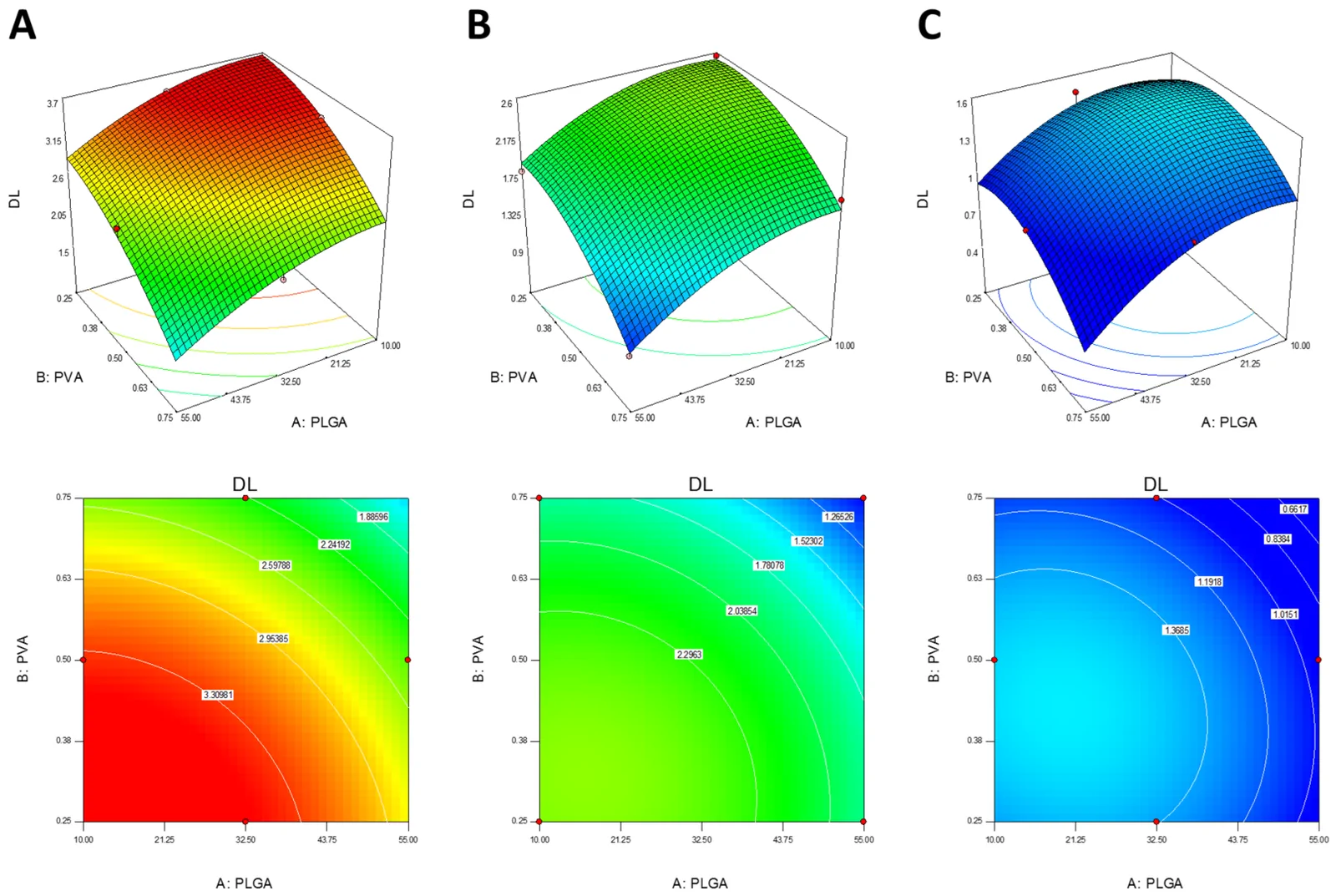
Surface plots (upper) and contour plots (lower) showing the effects of variables A and B on DL at a fixed amount of CHCl_3_: (**A**)—2.5 mL, (**B**)—5.0 mL, (**C**)—7.5 mL.

**Figure 5:**
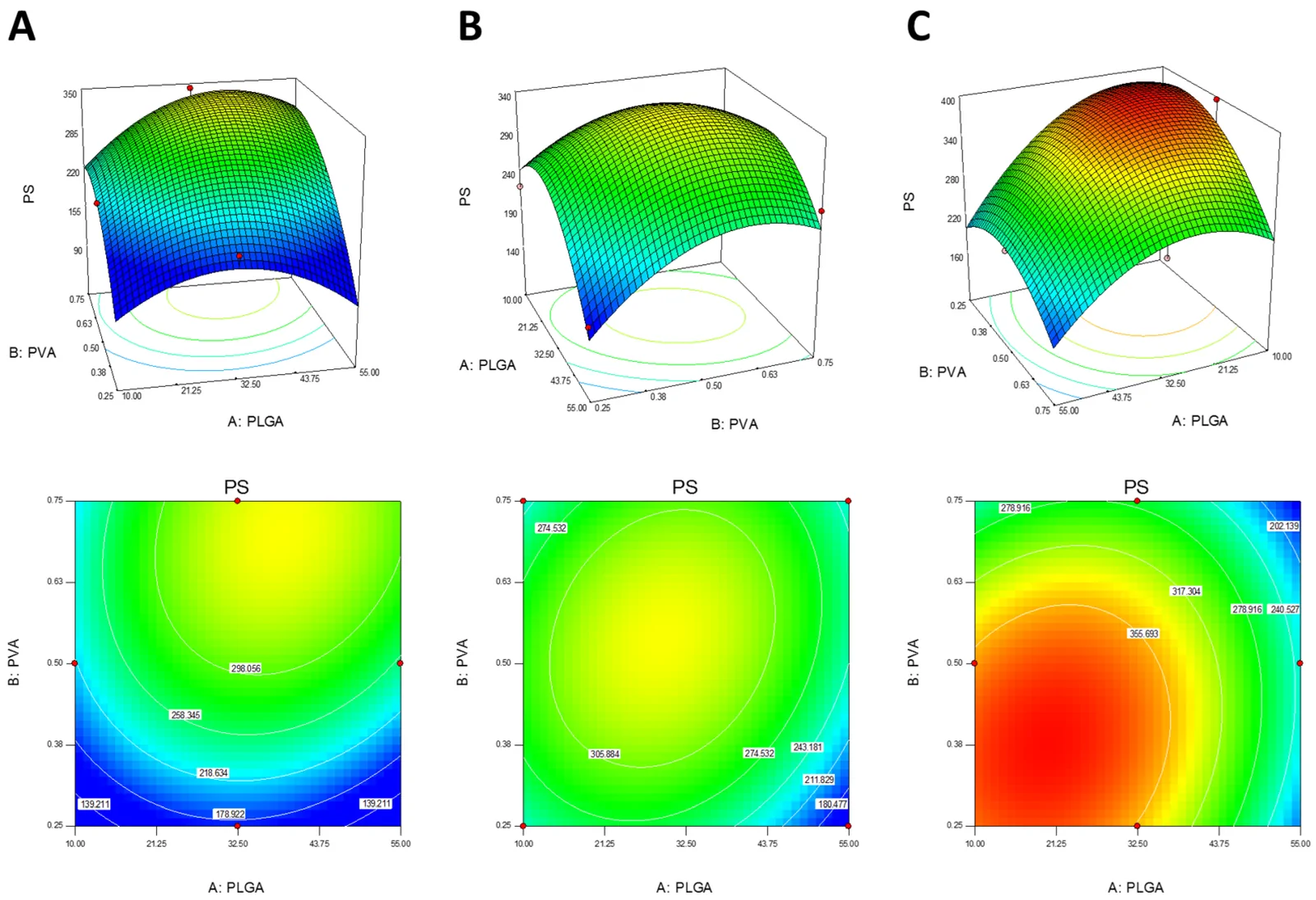
Two- and three-dimensional diagrams of PS response surfaces when varying the concentration of PVA and the amount of PLGA at a given amount of CHCl_3_: (**A**)—2.5 mL, (**B**)—5.0 mL, (**C**)—7.5 mL.

The response surface and contour plots presented in Figure 4 and Figure 5 depict the effects of two-variable interactions on DL and particle size at a fixed level of the third factor.

Having identified the significant factors and their interactions influencing the response surfaces, we performed numerical optimization to determine the optimal formulation conditions. We focused the optimization criteria on maximizing DL while minimizing size. From the solutions generated by the program, the option that was closest to optimal was chosen, as it had a higher probability of achieving the desired outcome. The calculated values of independent variables were applied to formulate optimized particles and analyze DL and size of COSAN-NPs. The optimal conditions corresponded to the desirability function value of 1.000 and to such parameters as 10 mg of PLGA, 0.33% PVA, and 2.77 mL of CHCl_3_. Based on optimal theoretical conditions, NPs were obtained and analyzed to obtain experimental DL and size value data (Table 3).

### 3.2. Preparation of COSAN-Loaded PLGA/PLGA-PEG NPs (NP-CPP)

Several studies have shown that the addition of polyethylene glycol (PEG) to PLGA enhances the incorporation of the drug into the polymer matrix [46,47], which is significant since high boron concentrations must be achieved for BNCT. It was also observed that the amphiphilic nature of PEG-PLGA provides a longer circulation time in the bloodstream, which is very important for enhancing the bioavailability of encapsulated drugs [48]. Hence, it was decided to prepare PLGA/PLGA-PEG NPs to increase COSAN DL (by UV-vis spectroscopy) and to study the effect of PLGA/PLGA-PEG ratio on the physicochemical characteristics of the obtained NPs. The PLGA-PEG copolymer was synthesized according to a previously developed scheme [41]. NPs based on different PLGA/PLGA-PEG ratios were prepared by the optimized technique, and their physicochemical analysis was performed (Table 4).

The ratio of 40% PLGA and 60% PLGA-PEG yielded appropriate values for PS, PDI, and ζ-potential, providing sufficient colloidal stability and monodispersed distribution. Therefore, this ratio was selected as the optimum ratio and was further used for the preparation of nanoparticles.

### 3.3. Morphology and FTIR Analysis

**Figure 6:**
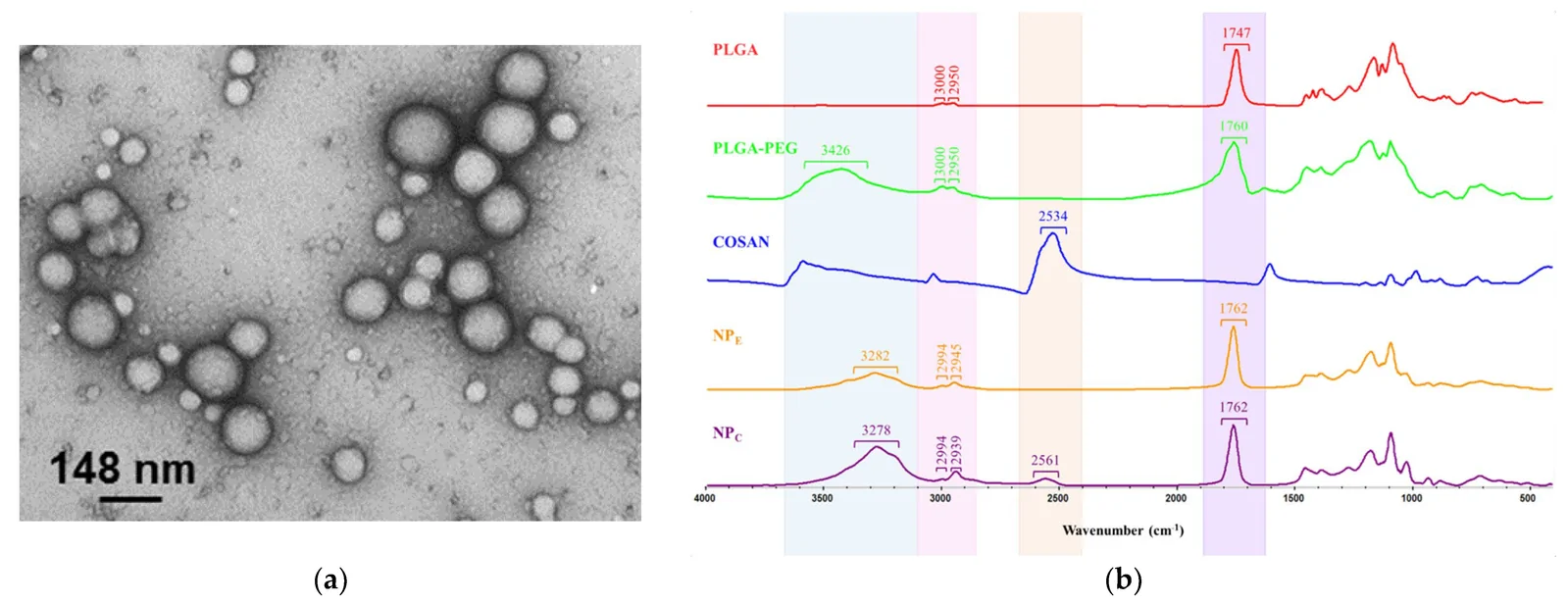
(**a**) TEM microphotograph of COSAN-loaded PLGA NPs: NP-CPP; (**b**) IR spectrum of COSAN, copolymers (PLGA and PLGA-PEG) and nanoparticles: NPE—empty nanoparticles without COSAN; NPC—nanoparticles containing COSAN.

We employed TEM to examine the morphology and particle size of NP-CPP. The TEM micrograph revealed well-defined, smooth spherical particles with a narrow size distribution and no visible aggregation (Figure 6a). FTIR was used to study the interaction between COSAN and PLGA (Figure 6b).

The absence of new peaks proves that the COSAN structure was intact after encapsulation into the polymer matrix.

### 3.4. Preparation of Cy5.5-Loaded NPs (NP-CPPC)

COSAN and Cy5.5 dual-loaded PLGA nanoparticles were prepared to investigate the accumulation of NPs in vitro and to evaluate the biodistribution in vivo. The optimized technology with a PLGA/PLGA-PEG ratio of 40:60 (*w*/*w* %) was used. UV-vis spectroscopy of NP-CPPC revealed the characteristic band of COSAN at 284 nm and maximum absorbance of Cy5.5 at 687 nm in DMSO, providing the simultaneous DL analysis of COSAN and Cy5.5 in NP-CPPC. According to the physicochemical analysis data, the obtained NP-CPPC particles had a PS of 166 ± 17 nm, PDI of 0.273 ± 0.038, ζ-potential of −21.8 ± 1.7 mV, and DL of 16.1 ± 0.3% for COSAN and 8.4 ± 0.1% for Cy5.5. Dual encapsulation slightly increased the PS and PDI of NP-CPPC compared to COSAN monoencapsulated NPs, the effect being observed previously [38]. Cy5.5 and COSAN co-encapsulation lacked any effect on ζ-potential, maintaining the value that provides sufficient colloidal stability. Thus, the obtained NP-CPPC possessed desirable parameters and was further used in experiments to study the accumulation in cells in vitro and to evaluate the biodistribution in vivo.

### 3.5. Drug Release Kinetics

**Figure 7:**
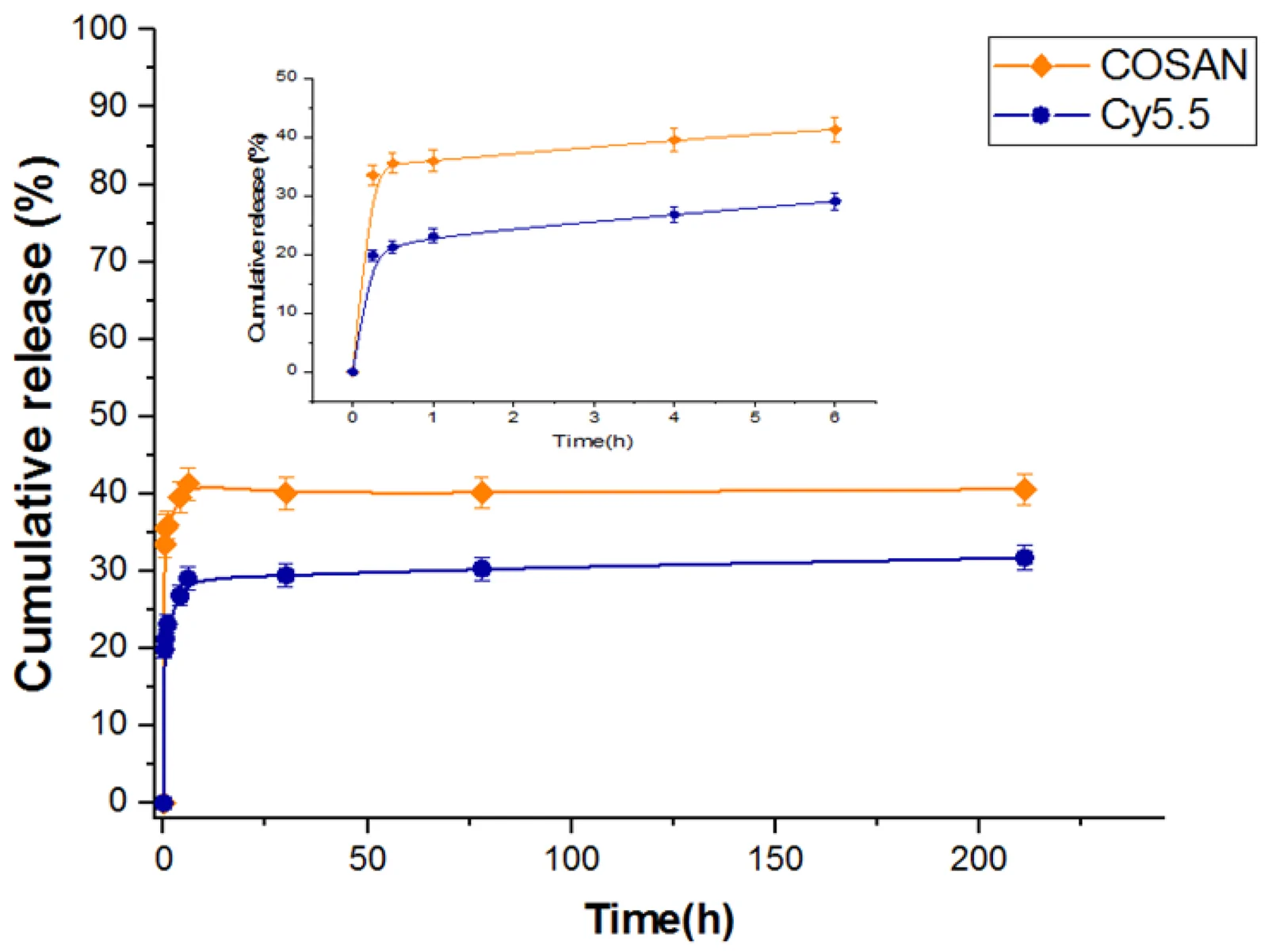
In vitro release profile of COSAN and Cy5.5 from NP-CPPC in 0.01 M PBS, pH 7.4, +37 °C. Each point shows mean ± S.D. (n = 3).

The kinetics of COSAN and Cy5.5 release from NP-CPPC showed a release of about 40% of COSAN and 31% of Cy5.5 from the polymer matrix in 211 h (Figure 7). In the case of both substances, a concentration spike—«burst»-effect—was observed in the first 6 h. Drug release was determined using UV-vis spectroscopy.

An assessment of the possible mechanism of drug release from polymer particles was carried out. To identify the most appropriate kinetic model, we fitted the release data to zero-order, first-order, Higuchi (H), Hixson–Crowell (H-C), Korsmeyer–Peppas (K-P), and Weibull (W) models and compared their goodness of fit using the coefficient of determination (R^2^) (Table 5).

The Weibull model achieved the most accurate approximation in all experiments.

### 3.6. Determination of NP-CPPC Hemolytic Activity

NP-CPPC hemolytic activity was studied to assess its hemocompatibility and possible undesirable changes in blood due to its exposure (Table 6).

Hence, NP-CPPC in concentrations above 4 mg/mL tended to exert hemolytic activity. Importantly, the concentrations below 4 mg/mL lacked hemolytic activity and were tested in further biological experiments.

### 3.7. Cytotoxicity and Accumulation of COSAN and NP-CPPC

**Figure 8:**
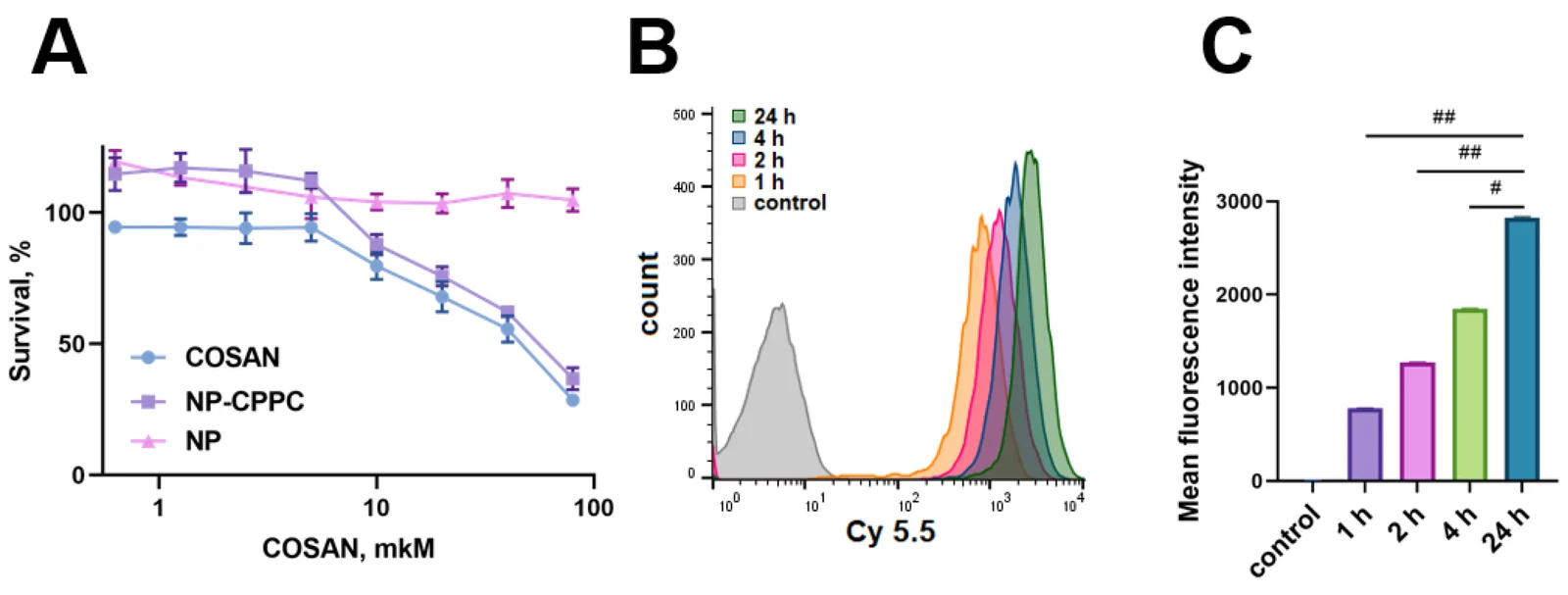
In vitro cytotoxicity of COSAN, blank NPs (NP) and NP-CPPC against B16 cells (**A**). Accumulation of NP-CPPC (50 μM) in B16 cells analyzed by flow cytometry (fluorescence histograms correspond to Cy5.5 co-encapsulated into nanoparticles) (**B**). Mean fluorescence intensity of Cy5.5 co-encapsulated into nanoparticles obtained by flow cytometry (**C**). The results are shown as the mean ± S.D. (n = 3). ^#^ *p* < 0.05 compared with the group under 4 h of NP-CPPC treatment; ^##^ *p* < 0.01 compared with the group under 1 h and 2 h of NP-CPPC treatment.

Since one of the main requirements for BNCT agents is low toxicity, the cytotoxic activity of the developed nanoparticles was evaluated and compared with COSAN itself and blank NPs (Figure 8A, Supplementary Materials, Figure S1).

**Figure 9:**
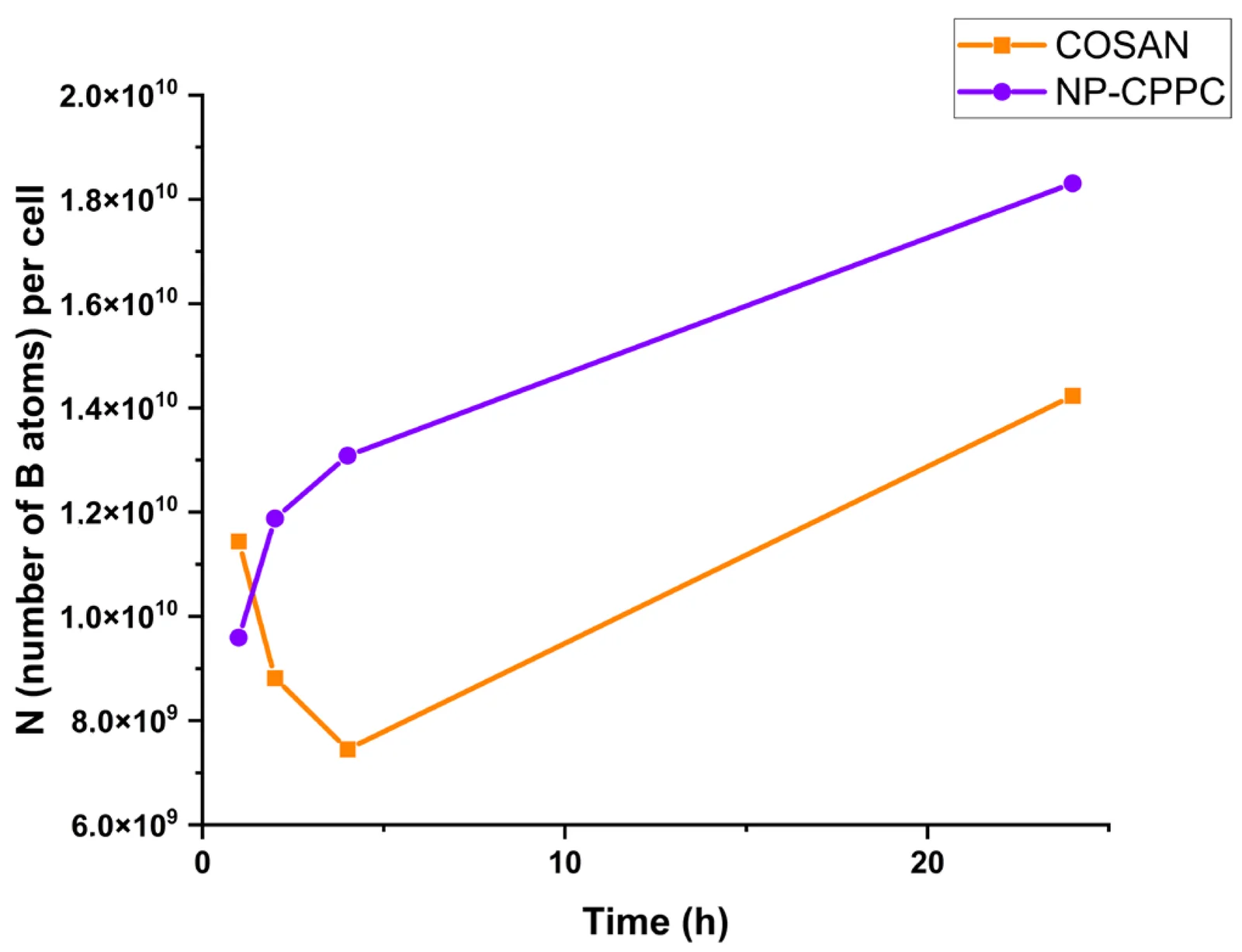
Results of NP-CPPC and COSAN accumulation in B16 cells.

Thus, NP-CPPC may be a promising candidate that fulfills the main requirements of low toxicity. Next, the accumulation of NP-CPPC in B16 cells was studied with ICP-MS analysis to ensure NPs deliver the required level of boron in vitro (Figure 9).

### 3.8. NP-CPPC Accumulation in B16 Cells by Laser Confocal Scanning Microscopy

**Figure 10:**
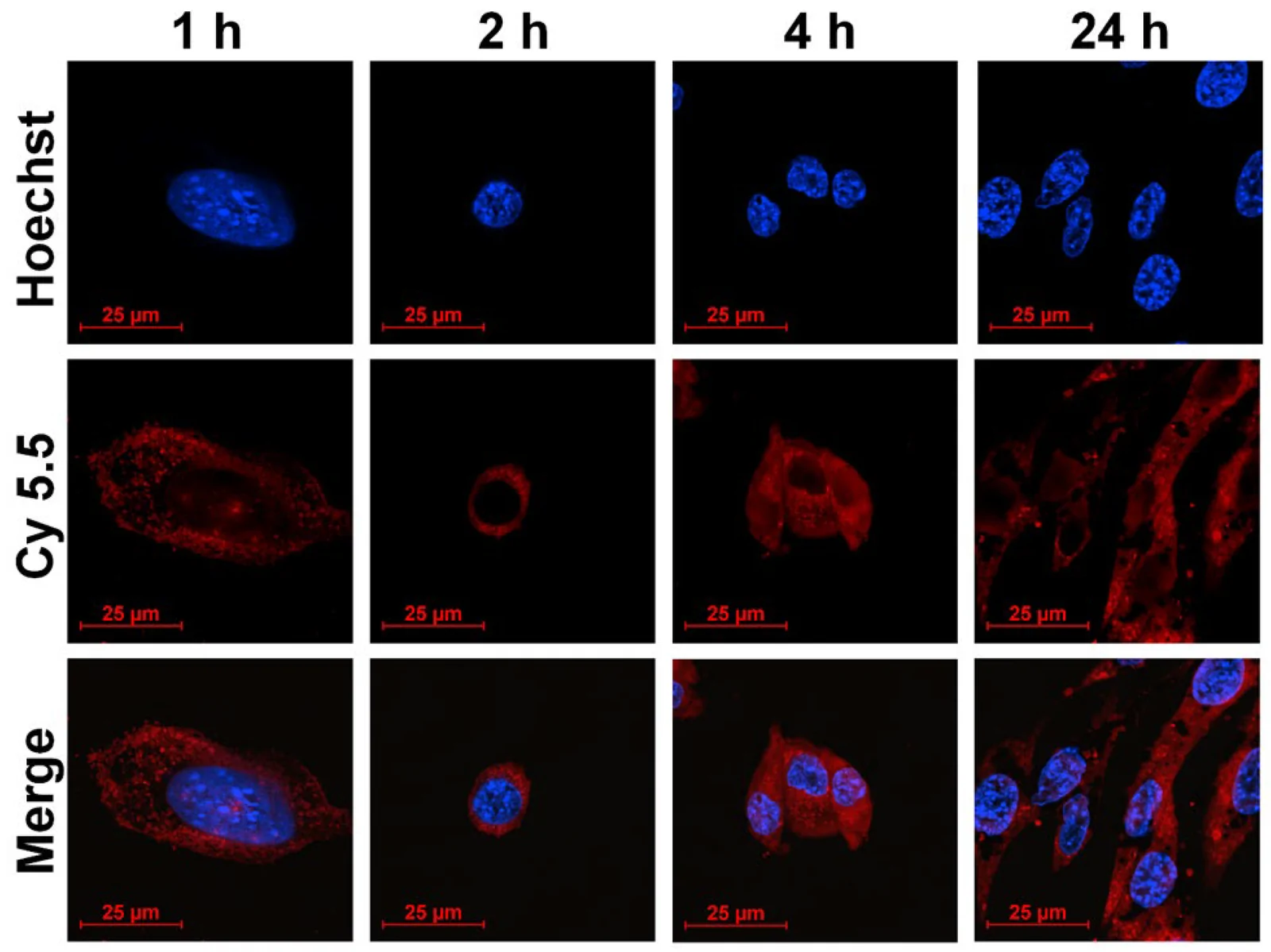
Laser confocal scanning microscopy images of B16 cells incubated with 50 μM NP-CPPC containing Cy5.5 (red). Subcellular localization was analyzed after treatment with Hoechst 34580 (blue). 25 μm scale bars.

The next important requirement for a BNCT agent is its sufficient concentration in the tumor, so NP-CPPC accumulation in B16 cells was assessed by flow cytometry, laser confocal microscopy and inductively coupled plasma mass spectrometry. For this purpose, B16 cells were incubated with NP-CPPC at a concentration equal to IC50 for 1, 2, 4 and 24 h. In order to visualize NPs in cells, we co-encapsulated COSAN and fluorescent probe Cy5.5 into PLGA nanoparticles (Figure 10). Cy5.5-associated red fluorescence was detectable in B16 cells at all investigated time points. In merged images, the red signal was predominantly extranuclear and spatially distinct from Hoechst-positive nuclei, consistent with cell-associated accumulation of the Cy5.5-labeled nanoparticle formulation in the cytoplasmic region. The fluorescence signal appeared heterogeneous and punctate within individual cells, and it remained readily detectable after 24 h of incubation. These confocal data provide qualitative evidence of sustained cellular association of NP-CPPC over the 24 h observation period.

To assess whether the polymeric carrier itself displayed a comparable cellular-association pattern, B16 cells were also incubated with the Cy5.5-labeled blank nanoparticles under the corresponding experimental conditions (Supplementary Materials, Figure S2). A similar internalization and cellular accumulation profile can be observed as for nanoparticles NP-CPPC.

### 3.9. NP-CPPC Accumulation in 3D Cell Culture Models

**Figure 11:**
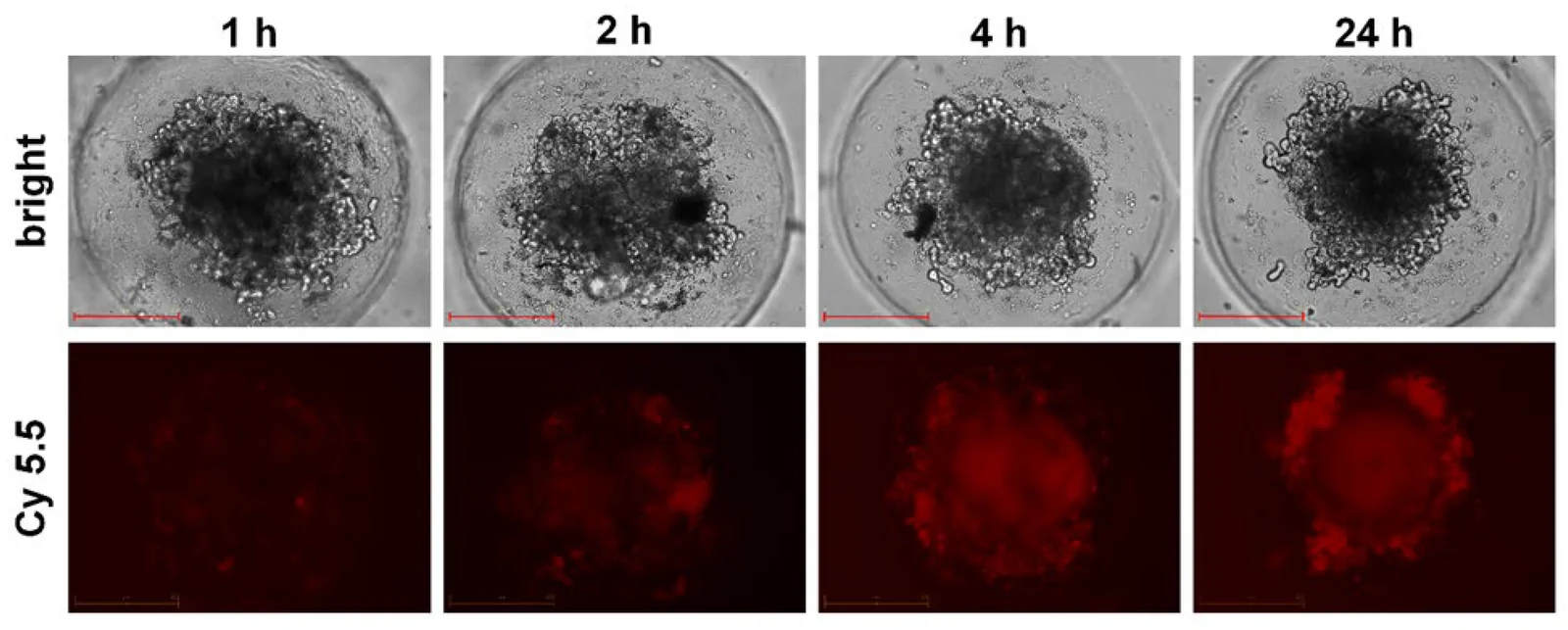
3D cultures of B16 cells treated with 50 μM NP-CPPC containing Cy5.5. Captured images from JuLI™ Stage imaging system using a ×10 objective. Scale bars: 250 μm.

3D B16 spheroids were used (Figure 11) as an intermediate model between conventional 2D monolayers and in vivo because they provide a compact multicellular architecture and permit assessment of nanoparticle-associated fluorescence in a more structurally complex model [49]. Cy5.5-associated fluorescence was detected following exposure to NP-CPPC and remained visible during the 24 h observation period. The red signal was heterogeneous across the spheroid area and was visually more prominent in peripheral and intermediate regions than in the optically dense central core. These findings are consistent with association and retention of the Cy5.5-labeled NP-CPPC formulation in the outer regions of B16 spheroids.

Cy5.5-labeled blank PLGA/PLGA–PEG nanoparticles were evaluated under the corresponding conditions as a carrier control (Supplementary Figure S3). The blank formulation showed a qualitatively similar heterogeneous fluorescence pattern, indicating that the PLGA/PLGA–PEG carrier itself can associate with the B16 spheroids.

### 3.10. In Vivo Biodistribution

**Figure 12:**
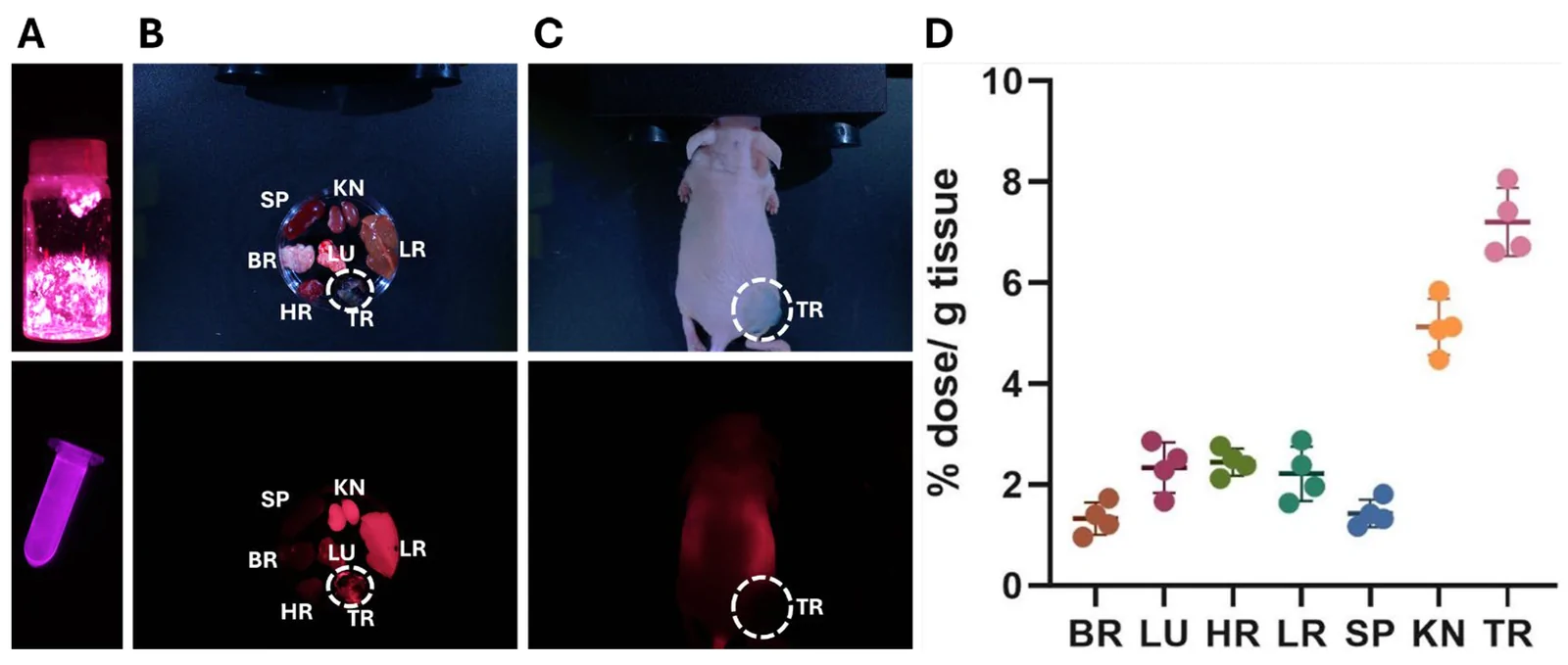
In vivo distribution of NP-CPPC in B16 tumor-bearing BALB/c nude mice. (**A**) Cy5.5 fluorescence images of dry (upper) NP-CPPC and resuspended in PBS (lower). (**B**) Representative bright-field (upper) and Cy5.5 fluorescence (lower) images of the excised B16 tumor and organs collected 12 h after intravenous administration. (**C**) Representative whole-body bright-field (upper) and Cy5.5 fluorescence (lower) images obtained 12 h after *iv* administration of 50 µg NP-CPPC in 100 µL PBS. The subcutaneous B16 tumor (~500 mm^3^; right flank) is indicated by the dashed line. Because B16 tumor melanin attenuates optical fluorescence and Cy5.5 is co-encapsulated rather than covalently linked to the carrier, panel C is qualitative. (**D**) Quantitative tissue-boron distribution measured by ICP-MS at 12 h (n = 4). Individual values and mean ± SD are shown. % injected total boron dose (ID)/g tissue. BR, brain; LU, lung; HR, heart; LR, liver; SP, spleen; KN, kidney; TR, tumor.

A subcutaneous B16 melanoma model was established in female BALB/c nude mice by inoculation of B16 cells. Once tumors reached a volume of approximately 500 mm^3^, animals received a single intravenous (i.v.) injection of 50 µg NP-CPPC. At 12 h after injection, the animals were subjected to in vivo live-fluorescence analysis and euthanized and the tumors were collected with the brain, lung, heart, liver, spleen, and kidney for ICP-MS analysis (Figure 12). In vivo Cy5.5 imaging showed a weak and spatially diffuse fluorescence signal in the melanotic tumor. Therefore, fluorescence was interpreted qualitatively, whereas tissue boron distribution was determined quantitatively by ICP-MS.

The mean tissue-normalized signals were 1.33 ± 0.32%ID/g in brain, 2.34 ± 0.50% ID/g in lung, 2.44 ± 0.27% ID/g in heart, 2.22 ± 0.54% ID/g in liver, 1.43 ± 0.27% ID/g in spleen, 5.13 ± 0.56% ID/g in kidney, and 7.20 ± 0.67% ID/g in tumor (Figure 12D). The paired tumor-to-kidney ratio was 1.41 ± 0.15.

Tumor-to-brain, tumor-to-lung, tumor-to-heart, tumor-to-liver, and tumor-to-spleen ratios were 5.66 ± 1.28, 3.16 ± 0.53, 2.97 ± 0.37, 3.40 ± 0.90, and 5.18 ± 1.08, respectively. However, normal skin, skeletal muscle, and blood were not collected in this preliminary study; therefore, tumor-to-local-normal-tissue ratios, except as mentioned (Table 7), could not be determined. A further limitation is that the intratumoral ^1^^0^B concentration achieved under the present intentionally low-dose regimen was 0.311 ± 0.029 µg ^1^^0^B/g tissue. This value is below commonly cited BNCT delivery benchmarks of approximately 10–20 µg ^1^^0^B/g tumor. The present experiment was designed to characterize preliminary tissue distribution after a low exploratory NP-CPPC dose, rather than to establish therapeutic dose or BNCT efficacy. The low 50 µg nanoparticle dose leaves scope for dose-escalation studies. Related blank PLGA-mPEG nanoparticles have been tolerated in mice at substantially higher intravenous doses [50] (Supplementary Materials, Figure S4). An important limitation is that no neutron-irradiation experiments were performed in the present study. Further study will evaluate dose–biodistribution relationships, single- and repeated-dose tolerability, multi-time-point pharmacokinetics, tumor-to-normal-tissue ratios, and neutron-irradiation responses.

## 4. Discussion

The COSAN-loaded PLGA nanoparticles were prepared via the double emulsion-solvent evaporation technique. Previous studies showed that size and drug loading influence the efficacy and safety of PLGA nanoparticles [51]. Increasing the drug loading within a delivery system enhances its desirability, as this can lead to a reduction in the frequency of drug administration and improve the concentration of the drug within target cells. The concentrations of PLGA and stabilizer are recognized as the primary determinants of drug loading in PLGA-based nanoparticles [52]. In addition to drug loading, particle size must fall within a defined range to achieve the desired pharmacokinetic profile; sizes between 50 and 250 nm are generally targeted to exploit the EPR effect [53]. Beyond polymer and stabilizer concentrations, solution viscosity also exerts a considerable influence on particle size [54].

We studied the influence of PVA concentration (%), amount of PLGA (mg) and volume of chloroform CHCl_3_ (ml) on DL (%) and particle size (PS) (nm) of COSAN-NPs via BBD. The R^2^ value for Equation 3 was 0.9916, reflecting a strong agreement between experimental and predicted values; R^2^ > 0.50 is considered acceptable [55]. The adjusted R^2^ of 0.9691 confirmed that the model accounted for the majority of the observed variance. The interactions between independent and dependent variables were further illustrated using 2D contour and 3D response surface plots (Figure 4 and Figure 5). The correlation between variables A and B has a pronounced effect on DL of the NPs at a lower CHCl_3_ amount (2.5 mL, Figure 4A). The highest value of DL, ranging from 3.60% to 3.65%, can be obtained with the smallest values of PLGA amount (10 mg–15 mg) and PVA concentrations (0.28–0.34%). At the middle level of CHCl_3_ amount (5.0 mL, Figure 4B), a similar dependence is observed, with the DL values increasing with decreasing PLGA amount and PVA concentration. In the case of the highest level of CHCl_3_ amount (7.5 mL, Figure 4C), for all values of PLGA amount and PVA concentration, DL of COSAN-NPs reaches its moderate values (below 1.5%). Figure 5 shows the influences of PVA concentration, PLGA amount, and amount of CHCl_3_ on the size of COSAN-NPs. Provided a constant amount of CHCl_3_, Figure 5A clearly exhibits an increase in PS with an increase in PVA concentration and negligible effect of PLGA amount variation. When the amount of CHCl_3_ increases (Figure 5B,C), varying the PLGA amount has a greater effect on particle size: the greater the PLGA amount, the smaller the size. The highest values of PS are achieved simultaneously with the increase of PVA concentration to the range of 0.38–0.63% (Figure 5B) and to the range of 0.27–0.60% (Figure 5C). Observed effects could arise due to increasing the ratio of the organic phase, which increases the viscosity of the emulsion and increases the size of the particle. As indicated in Table 3, the observed values were in close agreement with the predicted ones, verifying the BBD as an accurate and reliable approach for the optimization of the method of COSAN-NPs formulation. Next, we determined the PDI (0.148 ± 0.025) and zeta potential (−17.8 ± 4.1 mV) of the designed NPs. COSAN-NPs exhibited narrow size distribution (PDI < 0.2) and sufficient zeta-potential value for colloidal stability (>±10 mV) [51].

Varying the PLGA/PLGA-PEG ratio affected the size of NPs slightly, except when using 100% PLGA-PEG: PS was approximately two times higher compared to the others (Table 4). COSAN exhibited the highest DL value when using 40% PLGA and 60% PLGA-PEG. The amphiphilic nature of PLGA-PEG nanoparticles is a critical factor that enhances their effectiveness as drug delivery systems. This property arises from the distinct hydrophilic and hydrophobic segments within the block copolymer structure that allow for unique interactions with various drugs and biological environments. PLGA-PEG nanoparticles exhibit a self-assembled core–shell architecture in aqueous environments, where the hydrophobic PLGA constitutes the core, while the hydrophilic PEG forms the external shell. This structural arrangement facilitates the effective encapsulation of hydrophobic pharmaceuticals within the PLGA core, whereas hydrophilic drugs can associate with the PEG shell. This dual functionality significantly augments the overall drug loading efficiency by accommodating a broader spectrum of pharmaceutical agents, encompassing both hydrophobic and hydrophilic compounds [56,57].

The size value observed with TEM was smaller than that observed with DLS (Figure 6a). This can be explained by differences in the techniques: microscopy measures the gyration radius of dried NPs, while DLS measures the hydrodynamic diameter of NPs in suspension [58].

The FTIR spectrum of PLGA (Figure 6b) shows characteristic peaks at 1747 cm^−1^ (C=O vibrations), 2950 and 3000 cm^−1^ (C-H vibrations), which is in line with previous results [59]. The PLGA-PEG spectrum exhibits characteristic peaks at 1760 cm^−1^ (C=O vibrations), 2950 cm^−1^ and 3000 cm^−1^ (C-H vibrations), and 3426 cm^−1^ (N-H vibrations). In the COSAN spectrum, a characteristic peak at 2534 cm^−1^ (B-H vibrations) was observed. The icosahedral boron clusters (boranes, carboranes and metallacarboranes) show strong and characteristic ν(B-H) stretching in the infrared spectral range of 2600–2500 cm^−1^ where vibrations of other bonds are absent [12]. The spectra of NP_E_ and NP_C_ show a peak at 1762 cm^−1^ characterizing the C=O vibrations in the carboxyl group. The NP_C_ spectrum also shows a peak at 2561 cm^−1^ (B-H vibrations), indicating the encapsulation of COSAN into the nanoparticles. The spectra of NP_E_ and NP_C_ were similar to PLGA- PEG, except for the shift of the peak characterizing the N-H stretching from 3426 cm^−1^ to the region of 3282–3278 cm^−1^. The absence of new peaks proves that the COSAN structure was intact after encapsulation into the polymer matrix.

The kinetics of COSAN and Cy5.5 release from NP-CPPC showed the release of about 40% of COSAN and 31% of Cy5.5 from the polymer matrix in 211 h (Figure 7). In the case of both substances, a concentration spike—«burst»-effect—was observed in the first 6 h. Importantly, we observed similar COSAN and Cy5.5 release profiles and a negligible difference in final concentrations, which can be used to interpret in vitro and in vivo experiments to study nanoparticle biovisualization and accumulation. Next, we determined the mechanism of drug release from polymeric particles (Table 5). We used the coefficient of determination R^2^ to select the release kinetics models that fit the experimental data. Negative R^2^ values obtained for zero-order, first-order, Higuchi and Hixson–Crowell models indicate that the data did not adequately fit these models. Unlike linear regression, in nonlinear approximation, the R^2^ value is not restricted to the range from 0 to 1 and may take on negative values. This is because the coefficient of determination is calculated as:

R^2^ = 1 − (SSres/SStot), (5)

where SSres is the sum of the squares of the residuals, and SStot is the total sum of the squares.

The R^2^ value becomes negative when the SSres value exceeds SStot, meaning a mismatch between the selected model and the experimental data.

The Weibull model achieved the most accurate approximation in all experiments. We further analyzed the time exponent (β) of the Weibull function to assess the relationship between the exponent values and the probable mechanism of drug transport through the polymer matrix. The exponent β reveals the underlying mechanism by which a drug moves through a polymer matrix. A β value of 0.75 or less suggests Fickian diffusion. Values between 0.75 and 1 indicate anomalous or Case II transport, while values exceeding 1 point to super Case II transport [60]. Both COSAN and Cy5.5 demonstrated a β value less than 0.75 (0.025 for COSAN; 0.064 for Cy5.5), suggesting that diffusion was the dominant mechanism controlling their release, while the contribution of polymer swelling to the release kinetics was minimal. The data obtained from the experiment can be used to predict the release rate of COSAN from the nanoparticles, which makes it possible to calculate the optimal time for BNCT.

According to the value of hemolytic activity (Table 6), it is possible to judge the toxicity of the investigated drug delivery system in relation to blood cells. The greater the value of hemolysis indices of erythrocytes treated with the sample, the more toxic it is. According to ASTM E2524-22 [61], an increase in the percentage of hemolysis of more than 5% indicates red blood cell damage caused by the test materials. Hence, NP-CPPC concentrations above 4 mg/mL tend to exert hemolytic activity. Importantly, the concentrations below 4 mg/mL lacked hemolytic activity and were tested in further biological experiments.

It was earlier shown that COSAN demonstrates no relevant cytotoxic activity against normal cells, such as Chinese hamster pulmonary fibroblast V79 cells [11]. The results of the MTT test (Figure 8A) revealed that COSAN had weakly pronounced cytotoxic activity on B16 cells (44 μM) and COSAN incorporation in nanoparticles did not affect its activity (54 μM).

ICP-MS analysis (Figure 8B,C) revealed that the accumulation level of NP-CPPC gradually increased during 24 h of incubation with B16 cells. This could be attributed to favorable encapsulation effect of COSAN into NPs: the NPs inhibit rapid efflux of COSAN in cells and provide prolongated drug release. Oppositely, free COSAN tends to be rapidly eliminated from cells. Overall NP-CPPC demonstrated enhanced accumulation in B16 cells compared to free COSAN. Importantly, the concentration of boron atoms per cell exceeded the required minimum of 10^8^–10^9^, reaching, in the case of NP-CPPC formulation, 1.831·10^13^ after 24 h (Figure 9). By flow cytometry analysis, we observed time-dependent accumulation of NP-CPPC in B16 cells (Figure 8B,C). It was observed that the highest Cy5.5 accumulation occurred 24 h after incubation with NP-CPPC.

Using laser confocal scanning microscopy, no pronounced overlap of Hoechst (blue signal) and Cy5.5 fluorescence (red signal indicating NP-CPPC distribution) was observed, evidencing a slight NP-CPPC accumulation in cell nuclei (Figure 10). In the first 2 h after NP-CPPC treatment, Cy5.5 distribution may indicate NP-CPPC localization in late endosomes and lysosomes [62]. After 4 and 24 h, a perinuclear cytoplasmic distribution of NP-CPPC in B16 cells was observed. With increasing NP-CPPC incubation time, Cy5.5 fluorescence intensity in B16 cells increased, which is consistent with flow cytometry data.

Therefore, NP-CPPC accumulation in 3D cultures of B16 cells was evaluated (Figure 11). It is worth noting that no decrease in spheroid diameter was detected during 24 h of incubation, indicating low cytotoxic activity of NP-CPPC. As in 2D monolayer culture, for spheroids, a time-dependent accumulation of NP-CPPC was observed. In the first 2 h after NP-CPPC exposure, the fluorescence signal of Cy5.5 was observed mainly at the periphery of spheroids; in the next 4 h, an increase in the Cy5.5 fluorescence signal and its spread from the periphery to the center of the spheroid was revealed. After 24 h, strong fluorescence of Cy5.5 appeared at the periphery of the spheroid. Thus, it was found that NP-CPPC may have the ability to accumulate in 3D cell cultures.

Initially, we examined the potential of COSAN-loaded NPs for in vivo application, evaluating intratumoral accumulation. The in vivo experiment (Figure 12) was designed as an exploratory biodistribution study after administration of a deliberately low NP-CPPC dose (50 µg per mouse), rather than as a therapeutic-dose BNCT study. At 12 h, ICP-MS showed the highest mean tissue-normalized boron signal in the tumor (7.20 ± 0.67% ID/g, n = 4), whereas the kidney also showed a comparatively high value (5.13 ± 0.56% ID/g). The paired tumor-to-kidney ratio was 1.41 ± 0.15, while the tumor-to-lung, tumor-to-heart, tumor-to-liver, tumor-to-spleen, and tumor-to-brain ratios were 3.16 ± 0.53, 2.97 ± 0.37, 3.40 ± 0.90, 5.18 ± 1.08, and 5.66 ± 1.28, respectively. Thus, the data indicate higher tumor-associated boron than in the non-renal organs measured, but only modest selectivity relative to the kidney. Because normal skin, skeletal muscle, and blood were not collected in this preliminary experiment, the present data do not establish a tumor-to-adjacent-normal-tissue ratio. The weak Cy5.5 fluorescence in the B16 tumor should not be interpreted as a direct quantitative measure of nanoparticle accumulation. B16 tumors contain abundant melanin, which can attenuate fluorescence readout in intact tissue. In melanin-positive B16-derived tumors, fluorescence signal intensities were reported to be substantially lower than in comparable melanin-negative tumors, particularly with increasing imaging depth [63,64,65], while NPs solution and dry NPs themselves demonstrated bright Cy5.5 fluorescence (Figure 12A,B). Moreover, Cy5.5 was co-encapsulated with COSAN rather than covalently linked to the polymeric carrier; therefore, the in vivo fluorescence signal may not necessarily trace intact NP-CPPC. Thus, in vivo fluorescence analysis of melanin-rich tumors should be performed with caution. High-level boron accumulation in kidneys was observed as well, which is probably explained by the gradual release of COSAN from NPs; these results correlated with fluorescent analysis—probably, desorbed lipophilic Cy5.5 molecules were also excreted slowly by the kidneys. The hydrodynamic size of NP-CPPC (166 ± 17 nm) was substantially above the usual glomerular-filtration size range for intact nanoparticles; thus, direct renal filtration of intact NP-CPPC should not be assumed [66]. Figure 12B,D shows that boron-containing molecules could be excreted through the kidneys due to the low molecular weight, allowing effective glomerular filtration. The renal signal may arise from renal handling of released low-molecular-weight COSAN and/or Cy5.5, uptake of nanoparticle-associated material, or a combination of these processes. The present single-time-point experiment cannot distinguish these possibilities and should be emphasized as one of the limitations of the study. Unexpectedly for PLGA NPs, the liver, as well as the spleen, demonstrated low boron accumulation levels correlating with fluorescence analysis data. These results are likely explained by the release of Cy5.5 and COSAN. The efficient tumor accumulation and enhanced intratumoral boron retention observed for NP-CPPC cannot be explained by the EPR effect alone; COSAN likely possesses intrinsic properties that further promote intratumoral retention. The EPR effect is driven by increased tumor vascular permeability and favors delivery systems with prolonged systemic circulation, which enhances the probability of extravasation into tumor tissue. The tumor accumulation and intratumoral retention of NP-CPPC are therefore attributed to passive EPR-mediated targeting, combined with endocytosis-driven cellular internalization that limits efflux from target cells, as supported by the in vitro accumulation data. These combined mechanisms resulted in the selective boron enrichment in tumor tissue observed for NP-CPPC (Figure 12D). Thus, NP-CPPC are expected to have the potential to deliver a therapeutic effect and will be analyzed in further studies including dose escalation, plasma and urine sampling, multi-time-point analysis, and payload to define the pharmacokinetic basis of the renal signal. Irradiation experiments will then test the optimized formulation using appropriate controls.

## 5. Conclusions

In the present study, we successfully encapsulated COSAN within PLGA nanoparticles using the double emulsion-solvent evaporation technique. Firstly, we used BBD to evaluate the influence of the main variable factors (PVA, PLGA, CHCl_3_) on the size and DL of the nanoparticles. After NP optimization, the value of COSAN DL exceeded 10%, indicating a high level of encapsulation. NP-CPPC examination with FTIR confirmed COSAN stability in the polymeric matrix. COSAN exhibited prolonged release from NP-CPPC, which is desirable for enhanced cell accumulation.

The MTT assay results indicated that both COSAN and NP-CPPC exhibited a negligible cytotoxic effect on B16 cells, and NP-CPPC lacked a toxic effect on erythrocytes in a hemolysis study. These results ensure the safety profile of NP-CPPC. The nanoparticles were efficiently taken up by B16 cancer cells and localized in the perinuclear region. ICP-MS measurements showed enhanced uptake of boron via NP-CPPC compared to free COSAN. Similar to 2D monolayer culture, a time-dependent accumulation of NP-CPPC for spheroids was observed. Despite the mentioned limitations, preliminary in vivo studies on B16-bearing BALB/c nude mice revealed specific NP-CPPC accumulation in tumors, indicating NP-CPPC is expected to have the potential to deliver a therapeutic effect. The obtained results serve as a basis for further in vivo experiments using irradiation for BNCT.

## Figures and Tables

**Table 1:** BBD independent factors and levels data.

Independent Factors		Levels	
		Low	High
A	PLGA, mg	10	55
B	PVA, %	0.25	0.75
C	CHCl_3_, mL	2.5	7.5

**Table 3:** Comparison of the observed and predicted data for COSAN-loaded NPs obtained using BBD.

Conditions	Responses	Observed Data	Predicted Data	Bias *, %
A: 10 mg B: 0.33% C: 2.77 mL	R1, nm	220	163	−35.0
	R2, %	3.8	3.6	−5.8

* % Bias = [(predicted value-observed value)/predicted value]·100.

**Table 4:** Physicochemical characteristics of NP-CPP. Data are presented as mean ± S.D. (n = 3).

NP-CPP	PLGA, %	PLGA-PEG, %	PS, nm	PDI	ζ-Potential, mV	DL, %
1	0	100	483 ± 23	0.279 ± 0.042	−25.4 ± 3.7	9.8 ± 0.2
2	20	80	255 ± 10	0.248 ± 0.034	−21.3 ± 3.4	8.8 ± 0.2
3	40	60	152 ± 13	0.261 ± 0.027	−21.3 ± 3.4	11.7 ± 0.3
4	60	40	191 ± 22	0.273 ± 0.059	−22.8 ± 3.0	8.4 ± 0.1
5	80	20	164 ± 23	0.268 ± 0.031	−17.6 ± 7.26	10.5 ± 0.1
6	100	0	220 ± 27	0.148 ± 0.025	−17.8 ± 4.1	3.8 ± 0.2

**Table 5:** Model fitting data (each number indicate R^2^ values).

Substance	Mathematical Model					
	Zero Order	First- Order	Higuchi (H)	Korsmeyer–Peppas (K-P)	Hixson–Crowell (H-C)	Weibull (W)
COSAN	−5.1170	−4.7413	−3.1777	0.9870	−4.8821	0.9916
Cy5.5	−3.8402	−3.5961	−2.0412	0.9799	−3.6831	0.9882

**Table 6:** Hemolytic activity of designed NP-CPPC.

Concentration of NP-CPPC, mg/mL	Hemolytic Activity, %
4.00	5.0
0.40	0.9
0.04	0.1
negative control	0
positive control	100

**Table 7:** Quantitative biodistribution of designed NP-CPPC.

Tissue	%ID/g, Mean ± SD	Tumor/Tissue, Mean ± SD	^10^B, µg/g, Mean ± SD
Brain	1.32 ± 0.32	5.66 ± 1.28	0.057 ± 0.014
Lung	2.33 ± 0.50	3.16 ± 0.53	0.101 ± 0.022
Heart	2.44 ± 0.27	2.97 ± 0.37	0.105 ± 0.012
Liver	2.22 ± 0.54	3.40 ± 0.90	0.096 ± 0.023
Spleen	1.43 ± 0.27	5.18 ± 1.08	0.062 ± 0.012
Kidney	5.13 ± 0.56	1.41 ± 0.15	0.222 ± 0.024
Tumor	7.20 ± 0.67	1.00	0.311 ± 0.029

## Data Availability

The original contributions presented in this study are included in the article. Further inquiries can be directed to the corresponding author.

## Supplementary Materials

The following supporting information can be downloaded at: https://www.mdpi.com/article/10.3390/jfb17080403/s1.
