# Supplementary material for: Engineering Cobalt Bis(dicarbollide)-Loaded Polymeric Nanocarriers: From Controlled Fabrication to Drug Delivery for BNCT
Source: J Funct Biomater. 2026 Aug 14;17(8):403. doi: 10.3390/jfb17080403 (PMC13514490; doi:10.3390/jfb17080403)
Supplement: Supplementary file 1 [file jfb-17-00403-s001.zip › jfb-4442730-supplementary.pdf]

Supplementary materials

Engineering Cobalt Bis(dicarbollide)-Loaded Polymeric Nanocarriers: From Controlled Fabrication to Drug Delivery for BNCT

Maksim A. Klimenko [1,*], Maria B. Sokol [1], Mariia R. Mollaeva [1], Nikita G. Yabbarov [1], Ivan A. Gulyaev [1], Margarita V. Chirkina [1], Olga E. Kamaeva [1], Igor B. Sivaev [2], Anna A. Druzina [2], Vladimir I. Bregadze [2], Irina Yu. Nikolaeva [3], Andrey Yu. Bychkov [3], Vladimir A. Khvostikov [4], Zhanna P. Burmii [4], Sergei I. Obydennyi [5,6] and Elena D. Nikolskaya [1,*]

1 N.M. Emanuel Institute of Biochemical Physics of Russian Academy of Sciences, 119334 Moscow, Russia;
2 A.N. Nesmeyanov Institute of Organoelement Compounds, Russian Academy of Sciences, 119334 Moscow, Russia;
3 Faculty of Geology, GSP-1, Lomonosov Moscow State University, Leninskie Gory, 119991 Moscow, Russia;
4 The Institute of Microelectronics Technology and High-Purity Materials, Russian Academy of Sciences, 142432 Chernogolovka, Russia;
5 Dmitry Rogachev National Medical Research Center of Pediatric Hematology, Oncology and Immunology, 117198 Moscow, Russia;
6 Center for Theoretical Problems of Physicochemical Pharmacology, 119334 Moscow, Russia
* Correspondence: klimenko@sky.chph.ras.ru (M.A.K.); elenanikolskaja@gmail.com (E.D.N.)

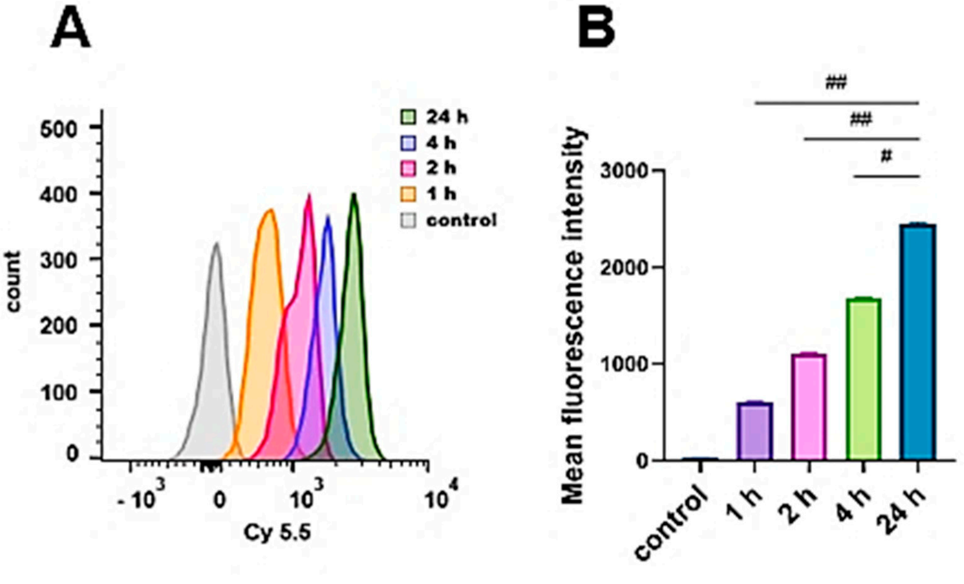

Figure S1. Accumulation of blank NPs (Cy 5.5) in B16 cells analyzed by flow cytometry (A). Mean fluorescence intensity of Cy5.5-encapsulated into blank nanoparticles obtained by flow cytometry (B). The results are shown as the mean ± S.D. (n = 3). #$p < 0.05$ compared with group under 4 h of NPs treatment; ##$p < 0.01$ compared with group under 1 h and 2 h of NPs treatment.

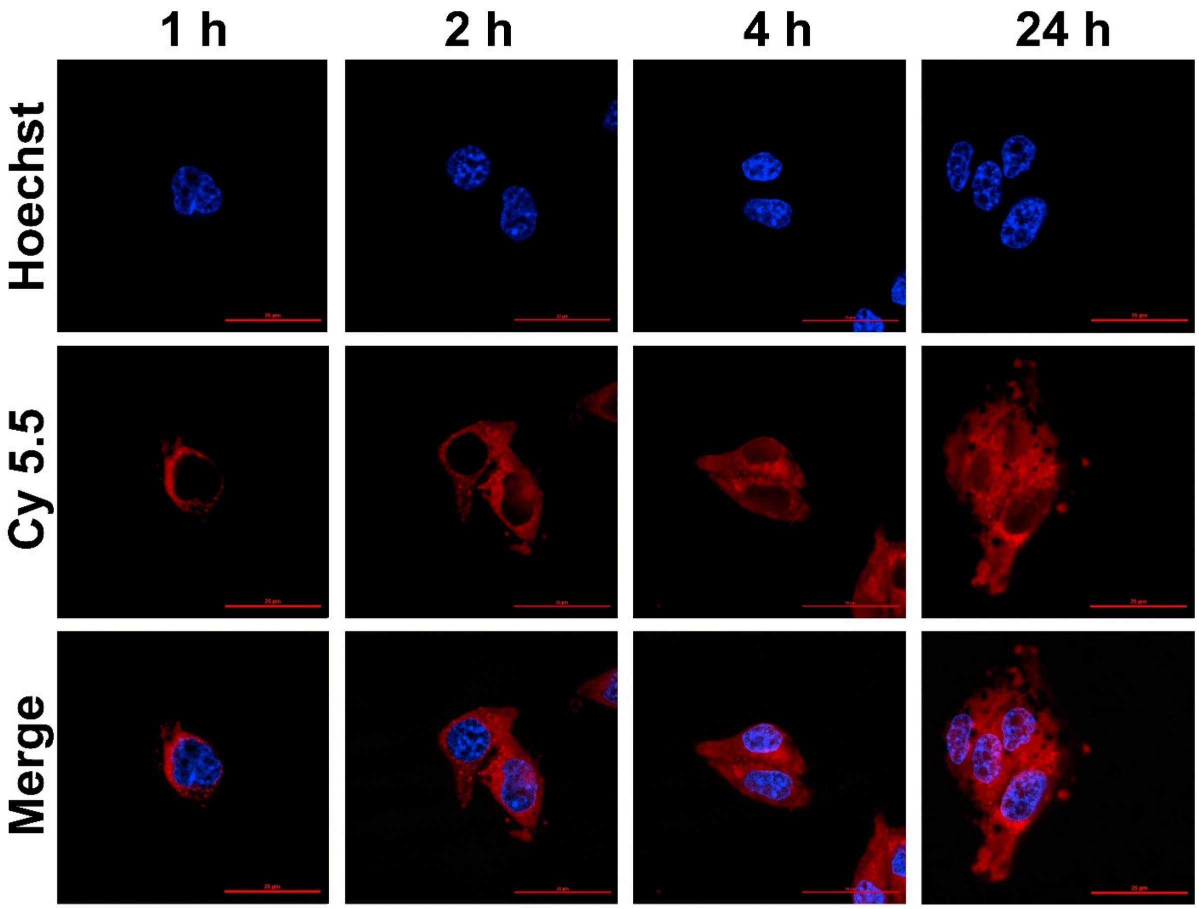

Figure S2. Laser confocal scanning microscopy images of B16 cells incubated with blank NPs containing Cy5.5. Subcellular localization was analyzed after treatment with Hoechst 34580. 25 μm scale bars.

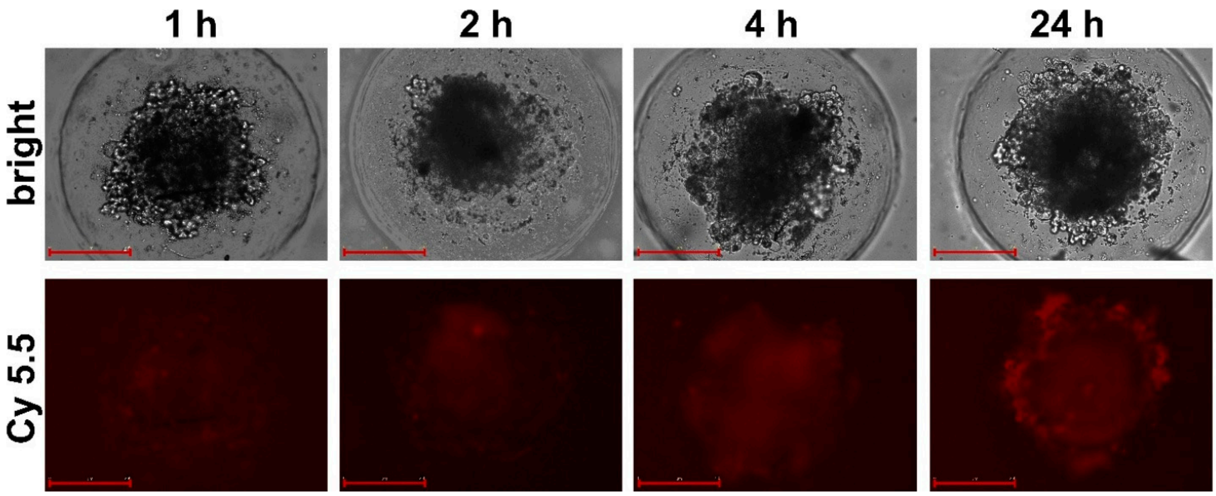

Figure S3. 3D cultures of B16 cells treated with blank NPs containing Cy5.5. Captured images from JuLI™ Stage imaging system using a ×10 objective. 250 μm scale bars.

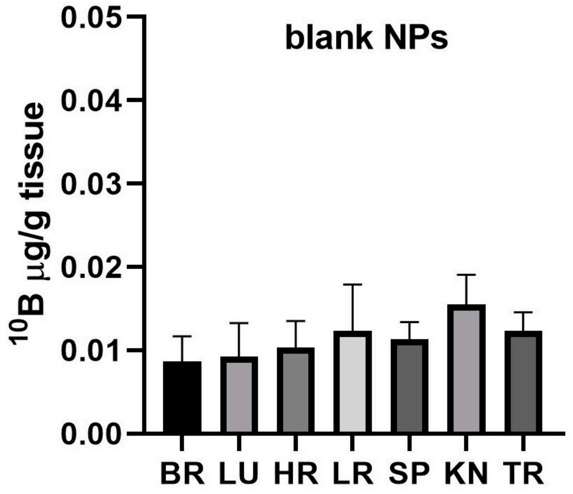

Figure S4. Tissue ^{10}B concentrations in B16 tumor-bearing mice after administration of blank PLGA/PLGA-PEG NPs. Blank nanoparticles matched the polymeric composition and 50 μg of the NP-CPPC formulation but contained no COSAN. Mice were euthanized 12 h after intravenous administration, and ^{10}B concentrations in brain (BR), lung (LU), heart (HR), liver (LR), spleen (SP), kidney (KN), and tumor (TR) were determined by ICP-MS. Values are presented as mean ± SD (n = 4). Because the blank formulation contained no COSAN, the low tissue values represent background ^{10}B.
